# Epiregulin is a dendritic cell-derived EGFR ligand that maintains skin and lung fibrosis

**DOI:** 10.1126/sciimmunol.abq6691

**Published:** 2022-12-09

**Authors:** Ian D. Odell, Holly Steach, Stephen B. Gauld, Lauren Reinke-Breen, Jozsef Karman, Tracy L. Carr, Joseph B Wetter, Lucy Phillips, Monique Hinchcliff, Richard A. Flavell

**Affiliations:** 1 Department of Dermatology, Yale University School of Medicine, New Haven, CT, USA; 2 Department of Immunobiology, Yale University School of Medicine, New Haven, CT, USA; 3 AbbVie Inc, North Chicago, IL, USA; 4 Department of Internal Medicine, Section of Rheumatology, Allergy & Immunology, Yale School of Medicine, New Haven, CT, USA; 5 Howard Hughes Medical Institute, Chevy Chase, MD, USA

## Abstract

Immune cells are fundamental regulators of extracellular matrix (ECM) production by fibroblasts and have important roles in determining extent of fibrosis in response to inflammation. Although much is known about fibroblast signaling in fibrosis, the molecular signals between immune cells and fibroblasts that drive its persistence are poorly understood. We therefore analyzed skin and lung samples of patients with diffuse cutaneous systemic sclerosis, an autoimmune disease that causes debilitating fibrosis of the skin and internal organs. Here, we define a critical role of epiregulin – EGFR signaling between dendritic cells and fibroblasts to maintain elevated ECM production and accumulation in fibrotic tissue. We found that epiregulin expression marks an inducible state of DC3 dendritic cells triggered by type I interferon and that DC3-derived epiregulin activates EGFR on fibroblasts, driving a positive feedback loop through NOTCH signaling. In mouse models of skin and lung fibrosis, epiregulin was essential for persistence of fibrosis in both tissues, which could be abrogated by epiregulin genetic deficiency or a neutralizing antibody. Notably, therapeutic administration of epiregulin antibody reversed fibrosis in patient skin and lung explants, identifying it as a previously unexplored biologic drug target. Our findings reveal epiregulin as a crucial immune signal that maintains skin and lung fibrosis in multiple diseases and represents a promising antifibrotic target.

## One Sentence Summary

Epiregulin is an EGFR ligand produced by DC3 dendritic cells that is essential for persistence of skin and lung fibrosis.

## Introduction

Pathologic fibrosis is a common final outcome of most human chronic inflammatory diseases and has been estimated to underlie almost half of all human deaths in the developed world (1). Despite the critical importance of fibrosis for wound healing, there is a major unmet medical need to identify effective antifibrotic therapies; yet our inability to precisely identify the dysregulated molecular circuits that drive fibrosis impedes drug development. Scleroderma (systemic sclerosis (SSc/scleroderma) is the prototypic human fibrotic disease, which most commonly affects the skin, but can also affect the lungs, kidneys, gastrointestinal tract, and heart. There are no US Food and Drug Administration (FDA)-approved therapies for SSc skin fibrosis and only two approved treatments for SSc lung fibrosis (nintedanib (2) and tocilizumab (3)) that merely slow, but do not reverse disease.

Receptor tyrosine kinases (RTK) such as PDGFRα (4) and FGFR3 (5) play an essential role in fibrosis because their activation in fibroblasts results in overexpression of extracellular matrix (ECM) gene products. EGFR is a member of the ERBB family of RTKs, is primarily expressed by epidermal keratinocytes and is aberrantly activated in solid cancers, such as lung and breast (6). In a recently identified SSc skin disease gene expression signature (the Scleroderma Skin Severity Score/4S), EGFR ligand expression correlated with skin fibrosis severity (7). However, direct EGFR inhibition has shown inconsistent results when tested in different mouse models of fibrosis. EGFR inhibition was reported to prevent skin, liver, and kidney fibrosis (8–10), but exacerbated lung fibrosis (11). These discrepant findings support the notion that hitherto unexplored signaling circuits and regulatory feedback loops, such as have been described between fibroblasts and macrophages in vitro (12), may play a central role in SSc-associated fibrosis.

Multiple studies have utilized single-cell RNA Sequencing (scRNA-Seq) to elucidate the lineages and origins of fibroblast populations in healthy and fibrotic skin (13–17). In recent years, increased attention has also been centered on the observation that innate immunity plays an important role in fibrosis (18), with important signals from a distinct subset of monocytes (19) and plasmacytoid dendritic cells (20). Skin biopsies from patients with SSc show dermal infiltration of CD14^+^ mononuclear cells, plasmacytoid dendritic cells and type 2 innate lymphoid cells (21–23). Fibroblasts in close proximity to mononuclear cell infiltrates show higher collagen gene expression in SSc skin (24, 25). These findings suggests that myeloid immune cells could drive fibroblast collagen production through currently undescribed mechanisms. We hypothesized that an immune-mesenchymal signaling circuit underlies SSc-related skin and lung fibrosis, and that targeting a specific ligand may prevent RTK activation of pathologic fibroblasts, yielding a more efficacious and better-tolerated therapeutic approach than the current limited options.

## Results

### EGFR activation marks pathogenic fibroblasts in SSc skin and lung

**Fig. 1.**
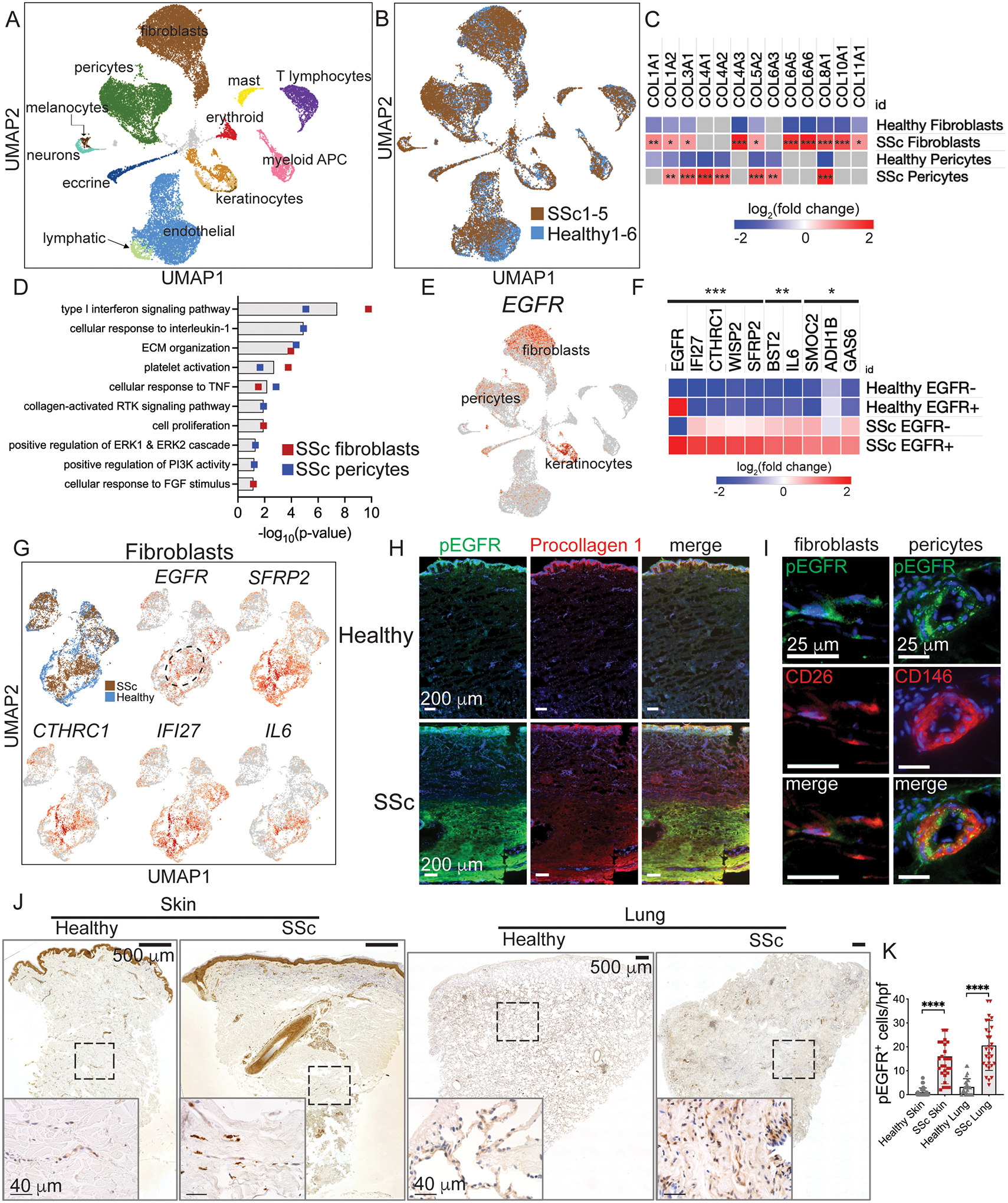
EGFR activation marks pathogenic fibroblasts in SSc skin and lung. (A,B) UMAP embedding of scRNA-seq data from five patients with diffuse cutaneous SSc and six healthy controls. (C) Heatmap of collagen gene expression in SSc fibroblasts and pericytes. (D) Gene ontology processes identified by upregulated genes in SSc fibroblasts and pericytes. (E) Expression of *EGFR* in UMAP embedded data. (F) Heatmap of upregulated genes in SSc *EGFR*-expressing fibroblasts compared to SSc *EGFR*-negative fibroblasts and healthy control fibroblasts. (G) UMAP plots of fibrotic gene expression by SSc and healthy fibroblasts. The dashed oval highlights *EGFR*^+^ SSc fibroblasts. (H, I) Immunofluorescence images of SSc skin co-stained with pEGFR and procollagen I, CD26 (DPP4), and CD146. (J) Low and high magnification photomicrographs of skin and lung from SSc and healthy subject samples stained with pEGFR antibody. Dashed boxes delineate regions shown in higher magnification image. (K) Enumeration of pEGFR^+^ cells in SSc skin dermis and lung (n=3 slides each, skin samples from patients SSc1, 3, and 4, 10 high power fields (hpf) per slide). Heatmaps in (C, F) are log_2_(fold change) of genes expressed by SSc vs healthy fibroblasts and pericytes (C) and SSc EGFR^+^ fibroblasts compared to SSc EGFR^−^ fibroblasts and EGFR^+^ and EGFR^−^ healthy fibroblasts (F) using sSeq and edgeR methods with p-values adjusted using the Benjamini-Hochberg correction for multiple tests. Data in (K) are means ± SD analyzed with unpaired two-tailed Student t test. For heatmaps and graphs, *=P<0.05, **=P<0.01, ***=P<0.001, ****P<0.0001. Slides were imaged with a Keyence BZ-X800 microscope. Low power images are 10x magnification and stitched together. High power images are 40x magnification.

To understand the cellular signaling that occurs in SSc skin, we obtained biopsies of fibrotic skin from five patients with diffuse cutaneous SSc and six healthy controls for scRNA-Seq. Clinical characteristics of the study participants including age, sex, affected organs, comorbid conditions, and immunomodulatory therapies at the time of biopsy are listed in Table S1. Immediately after skin biopsy, we digested the tissue, sorted all live cells, and used the 10x Chromium Single Cell Controller to create barcoded single-cell cDNA libraries. Clustering and Uniform Manifold Approximation and Projection (UMAP) embedding of the single cell cDNA identified 12 major cell clusters (Fig. 1a) each defined by a set of signature genes (Fig. S1a). Cells from individual SSc samples (colored brown) localized in similar regions of the fibroblast, pericyte and endothelial clusters (Fig. 1b), suggesting similar gene expression profile in the cells from fibrotic skin compared to those from healthy skin. As SSc is defined by the overabundance of ECM, we looked for cell types that showed differential expression of ECM genes. We found that SSc fibroblasts showed significantly increased expression of multiple ECM genes, including types I, III and additional collagens (Fig. 1c). Similarly, SSc pericytes, marked by their expression of *RGS5* (26), showed increased expression of collagen I, III, IV, and VI transcripts. Therefore, ECM in SSc is overexpressed by both fibroblasts and pericytes and our overarching goal was to identify immune factors that regulate this.

To identify signaling pathways that drive higher ECM production in SSc, we calculated the differential gene expression of each cell type in SSc compared to healthy controls. We performed functional enrichment analysis using DAVID (27) of upregulated genes with log2(fold change)>0.58 and p-value<0.05. Enriched gene ontology terms in SSc fibroblasts and pericytes versus healthy controls included expected results such as ECM organization and type I interferon signaling (28, 29) (Fig. 1d, full list in Data file S1). Moreover, we identified multiple pathways associated with receptor tyrosine kinase (RTK) signal transduction, including cell proliferation, response to FGF stimulus, collagen activated RTK signaling, positive regulation of ERK1 and ERK2 cascade, and positive regulation of PI3K activity. We focused on EGFR as one such RTK because multiple EGFR ligands are expressed by immune cells (discussed in the next section) and previous studies showed inhibition of EGFR to prevent fibrosis in mice (8–10). We thus interrogated our scRNA-Seq data to determine the cell types that most highly express EGFR. We found that EGFR is not only expressed by epidermal keratinocytes, but by fibroblasts and pericytes, the cells of interest that we observed to overexpress ECM genes (Fig. 1e). Thus, pathway analysis of our single cell data suggested RTK signaling is activated in SSc, and we identified EGFR as a promising candidate receptor because of its expression in both fibroblasts and pericytes.

We hypothesized that if EGFR-expressing (EGFR^+^) fibroblasts and pericytes are relevant to SSc pathogenesis, they would show differential expression of SSc gene signatures. Using our skin scRNA-Seq data, we calculated the differential gene expression of EGFR^+^ SSc fibroblasts compared to EGFR^−^ SSc fibroblasts and EGFR^+^ and EGFR^−^ healthy control fibroblasts. The upregulated genes with the highest differential expression and lowest p-value by EGFR^+^ SSc fibroblasts compared to the other fibroblast subsets include several markers of myofibroblasts and other fibrosis signature genes (Fig. 1f). Closer examination of fibroblasts demonstrated a specific region with this SSc gene signature (Fig. 1g). In particular, EGFR^+^ SSc fibroblasts expressed higher levels of genes associated with Wnt signaling (*SFRP2*, *CTHRC1*, and *WISP2*), a pathway activated by fibroblasts that mostly reside in the reticular dermis (13) and is hyperactivated in SSc skin (30). Among Wnt genes, *SFRP2* expression defines a subset of dermal fibroblasts (31, 32) that were recently shown to differentiate into myofibroblasts in SSc skin (15). *CTHRC1* (collagen triple helix repeat containing 1) is a gene whose expression defines pathogenic fibroblasts in SSc and idiopathic pulmonary fibrosis (IPF) fibrotic lung specimens (33) and is a marker along with *LRRC15* for myofibroblasts across tissues (16). EGFR^+^ SSc fibroblasts also expressed higher levels of interferon inducible genes (*IFI27*, *BST2*), which have been shown to correlate with SSc disease severity (34, 35), and *IL6*, a profibrotic cytokine whose inhibition slows SSc-associated lung fibrosis (3). In contrast to fibroblasts, SSc pericytes showed differential expression of multiple fibrotic genes compared to healthy control samples but were not distinguished by expression of EGFR. Thus, EGFR^+^ SSc fibroblasts, but not pericytes, express multiple pathologic signature genes associated with SSc skin and lung fibrosis.

We next determined where and in what cell types EGFR is activated in SSc fibrotic tissue. Upon activation, EGFR becomes phosphorylated at multiple sites on its cytoplasmic domains (36), detectable using phospho-specific antibodies to phosphorylated EGFR (pEGFR). To measure where activation of EGFR occurs in SSc, we stained skin from patient SSc1, who among our patients had the most recent onset of disease, with a phospho-specific antibody against EGFR Tyr-1068. To link EGFR activation with collagen production, we performed immunofluorescence imaging of pEGFR and procollagen I. From low power, pEGFR and pro-collagen co-stain SSc fibrotic dermis (Fig. 1h) and this was absent in healthy control skin as well as with the pEGFR isotype antibody (Fig. S1b). The pEGFR and procollagen I staining in the lower dermis matched the patient’s pattern of fibrosis on histology (Fig. S2a, 2^nd^ panel from the left) and lack of fibrosis and pEGFR signal in the upper dermis may be due to his relatively early disease. Imaging of pEGFR with markers of profibrotic fibroblasts (CD26 / DPP4) and pericytes (CD146 / MCAM) demonstrated that EGFR is activated on both fibroblasts and pericytes (Fig. 1i). According to our scRNA-Seq data, CD26 is also expressed by some T cells, lymphatic endothelial cells, and keratinocytes, which have different morphology and tissue location than the CD26^+^ cells shown in Fig. 1i. Myofibroblasts and pericytes, but not healthy fibroblasts, express α-smooth muscle actin (α-SMA), which we also found co-labeled with pEGFR (Fig. S1c). Thus altogether, EGFR is activated on myofibroblasts and pericytes in SSc skin.

We next quantified the number of pEGFR^+^ cells in SSc fibrotic tissue. We observed strongly labelled cells adjacent to and coursing within fibrotic dermis and within fibrotic lung tissue (Fig. 1j, isotype controls in Fig. S1d, and low power scans of histology and immunohistochemistry in Fig. S2). Quantification of pEGFR^+^ cells in skin and lung showed that they were significantly increased in SSc versus healthy control samples (Fig. 1k). Therefore, EGFR is activated in SSc fibroblasts and pericytes, which are located in SSc fibrotic skin and lung, further implicating EGFR activation as a marker of pathogenic fibroblasts in SSc.

### EREG^+^ dendritic cells accumulate in human skin and lung fibrosis

EGFR has seven activating ligands with different signaling properties based on their binding kinetics (37, 38). To characterize activating ligands of EGFR and other RTK in SSc skin, we identified receptor-ligand enrichment in our skin scRNA-Seq dataset using CellphoneDB (39, 40), which identifies increased expression of receptor-ligand pairs between cell clusters (https://www.cellphonedb.org). Significant interactions as indicated by rank < 0.05 in SSc vs healthy skin were identified and examined based on the cell types producing each ligand-receptor pair. Enriched interactions in the SSc skin signaling network were largely comprised of growth factor ligands and their receptors (depicted in Fig. S3a), including EGFR, PDGFR, NOTCH, Ephrins, and other receptor tyrosine kinases, with most growth signals occurring between mesenchymal cell types. Compared to mesenchymal cells, immune cells expressed distinct growth factor ligands including EGFR activating ligands and oncostatin M. These findings suggest that immune-mesenchymal and mesenchymal-mesenchymal growth factor signals support the cell-cell communication network in fibrotic skin.

**Fig. 2.**
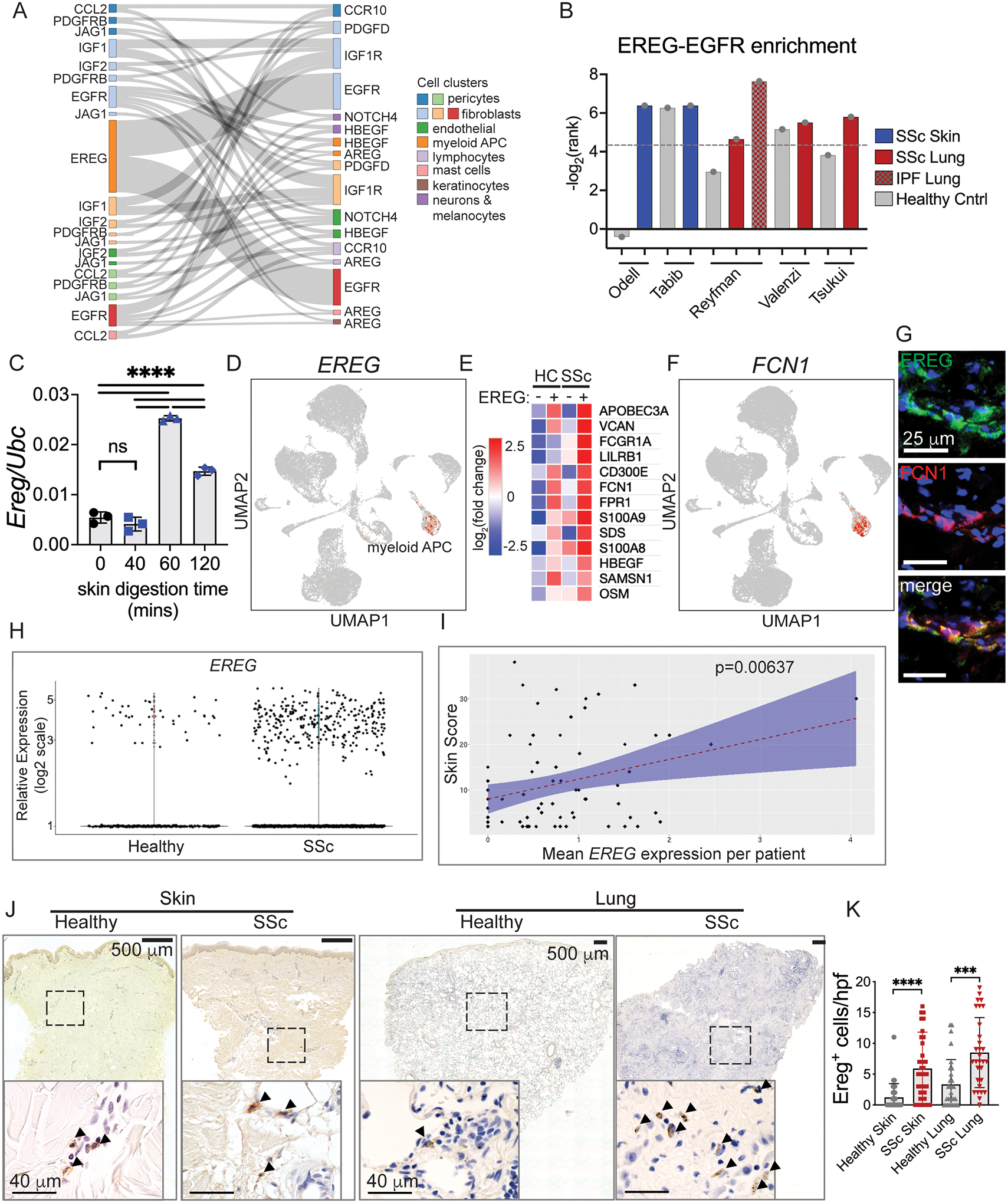
EREG^+^ dendritic cells accumulate in human skin and lung fibrosis. (A) Sankey diagram of enriched receptor-ligand pairs in SSc skin and at least two lung scRNA-Seq datasets. Ribbon width is proportional to 1/rank of the skin SSc data. (B) Plot of the CellphoneDB ranks (adjusted p-values) of the interaction of *EREG* with *EGFR* in our skin scRNA-Seq data as well as our analysis of available data from SSc skin (15) and lung (33, 41, 42). Dotted line shows rank = 0.05. (C) *Ereg* relative expression during a time course of tissue digestion of healthy mouse skin, n=3 per time point. (D) Expression of *EREG* in our UMAP embedded scRNA-Seq data. (E) Heatmap of co-expressed genes by SSc *EREG*-expressing APC (EREG^+^) compared to healthy EREG^+^ APC and EREG^−^ APC groups. For clarity, the raw gene list was filtered to genes primarily expressed by immune cells. (F) Expression of *FCN1* in our UMAP embedded scRNA-Seq data. (G) Immunofluorescence images of EREG and FCN1 in SSc skin. (H, I) Analysis of *EREG* expression in SSc compared to healthy controls (H) and compared to modified Rodnan Skin Score (mRSS) (I) using data from (49). (J) Low and high magnification photomicrographs of skin and lung from SSc and healthy subject samples stained with EREG antibody. Dashed boxes delineate region shown in higher magnification image. Arrowheads label positive cells. (K) Enumeration of EREG^+^ cells in SSc skin dermis and lung (n=3 slides each, skin samples from patients SSc1, 3, and 4, 10 high power fields (hpf) per slide). Slides were imaged with a Keyence BZ-X800 microscope. Low power images are at 10x magnification and stitched together. High power images are 40x magnification. Data are means ± SD (***P<0.001, ****P<0.0001) analyzed with one-way analysis of variance (ANOVA) with Tukey multiple-comparisons test (C) and unpaired two-tailed Student t test (K).

To investigate whether important interactions identified in SSc skin are reproducible in other tissues, we compared our skin scRNA-Seq data to publicly available scRNA-Seq data from another study of SSc skin (15) and three studies of SSc-associated pulmonary fibrosis of patients at the time of lung transplant (33, 41, 42). We visualized enriched interactions common to SSc skin and at least two of the lung datasets (Fig. 2a; full list including non-overlapping interactions provided in Data file S2). The most significant shared interaction among SSc skin and lung was between epiregulin (*EREG*) from the cluster of myeloid antigen presenting cells (APC) and EGFR in two subclusters of fibroblasts. We confirmed the expression of *EREG* was elevated in whole tissue of SSc skin compared to healthy controls by qPCR (Fig. S3b). Among myeloid APC, expression of the EGFR ligands *AREG* and *HBEGF* was enriched in the scRNA-Seq data to a lesser degree, but not increased when assayed by qPCR. To visualize the hierarchy of myeloid APC–fibroblast interactions, the rank of the EREG-EGFR interaction from each study was plotted (Fig. 2b). We observed enrichment of the EREG-EGFR interaction in all SSc datasets and its absence in most of the healthy control data. We determined that tissue digestion times for an hour or more, as done in the studies with EREG-EGFR enrichment in healthy controls (15, 42), induced EREG expression in healthy skin (Fig. 2c). This finding likely explains why studies with longer digests found higher EREG expression in their healthy samples. We also observed enrichment of EREG-EGFR in the lungs of patients with idiopathic pulmonary fibrosis (IPF), a related but pathologically distinct fibrotic lung disease. Altogether, these data suggest that elevated expression of *EGFR* and *EREG* occurs in multiple fibrotic diseases of the skin and lung.

The cellular origin and function of *EREG* has not clearly been defined. Among cell types in our skin scRNA-Seq data, *EREG* was only expressed in the cluster of myeloid APC, which likely includes dendritic cells, monocytes and macrophages (Fig. 2d). By FACS analysis of peripheral blood from healthy volunteers, we observed surface expression of EREG primarily on CD1c^+^ dendritic cells, indicating an expression pattern restricted to dendritic cells (Fig. S3c, d). We therefore investigated recent scRNA-Seq analysis of circulating immune cells, which divided CD1c^+^ dendritic cells into distinct subsets, termed DC2 and DC3 (43). In-depth analysis of DC2 vs DC3 showed expression of *EREG* particularly by the DC3 subset of dendritic cells (44). DC3 are considered an inflammatory subset of dendritic cells (45) derived from a distinct precursor than conventional dendritic cells and whose development depends on GM-CSF (46). In our skin data, *EREG*-expressing (EREG^+^) APC in SSc samples showed elevation of characteristic markers of DC3, including *FCN1* (p=4.78E-08) and *VCAN* (p=1.20E-14), interferon response (*APOBEC3A*, p=9.08E-16), and higher expression of the alarmins *S100A9* (p= 6.15E-07) and *S100A8* (p=3.24E-05) compared to healthy EREG^+^ APC and SSc and healthy EREG^−^ APC (Fig. 2e). Expression of the DC3 marker *FCN1* is limited to myeloid APC (Fig. 2f) and co-stains with EREG in SSc skin (Fig. 2g). Elevated expression of alarmins links inflammation and fibrosis (e.g. (47)) and correlates with dcSSc severity in patients (48), supporting EREG^+^ APC in SSc pathogenesis as part of a tissue damage response. Therefore, EREG^+^ APC in the skin match the expression profile described for DC3 in blood and express higher levels of alarmins which are associated with SSc severity.

Because *EREG* expression correlated with other markers of SSc severity, we interrogated whether *EREG* itself is associated with extent of SSc skin fibrosis. Since we did not have enough samples in our dataset to address this question, we analyzed the large-scale SSc dataset recently published (49). We found increased number of EREG^+^ cells in SSc skin compared to healthy controls (Fig. 2h) and that *EREG* expression showed a significant positive correlation with disease severity by modified Rodnan Skin Score (mRSS) (Fig. 2i). The strength of the correlation between EREG expression and mRSS was small to medium (Pearson correlation coefficient = 0.31). This correlation is likely underestimated because the longer skin digestion time (1 hour) they used in their protocol induces *EREG* expression. Nevertheless, *EREG* expression by DC3 is associated with severity of skin fibrosis in SSc.

If EREG^+^ dendritic cells activate EGFR on fibroblasts in SSc skin and lung, we expected they should accumulate in those tissues. We assessed the spatial localization and abundance of EREG^+^ dendritic cells in the SSc fibrotic skin and lung by immunohistochemistry and immunofluorescence. In SSc skin, we observed clusters of EREG^+^ dendritic cells perivascularly and located at the margins of fibrotic dermis, whereas in the lung they appeared to have greater density within fibrotic parenchyma and were scarce in healthy skin and lung (Fig. 2j). Moreover, enumeration of EREG^+^ dendritic cells from SSc skin and lung showed significantly increased numbers of EREG^+^ cells in each tissue compared to healthy controls (Fig. 2k). Thus, EREG^+^ dendritic cells localize to SSc fibrotic skin and lung and are increased in abundance in both tissues, suggesting they could be responsible for activating EGFR to drive disease.

### Interferon-EGFR-NOTCH axis regulates expression of *EREG* and ECM genes

**Fig. 3.**
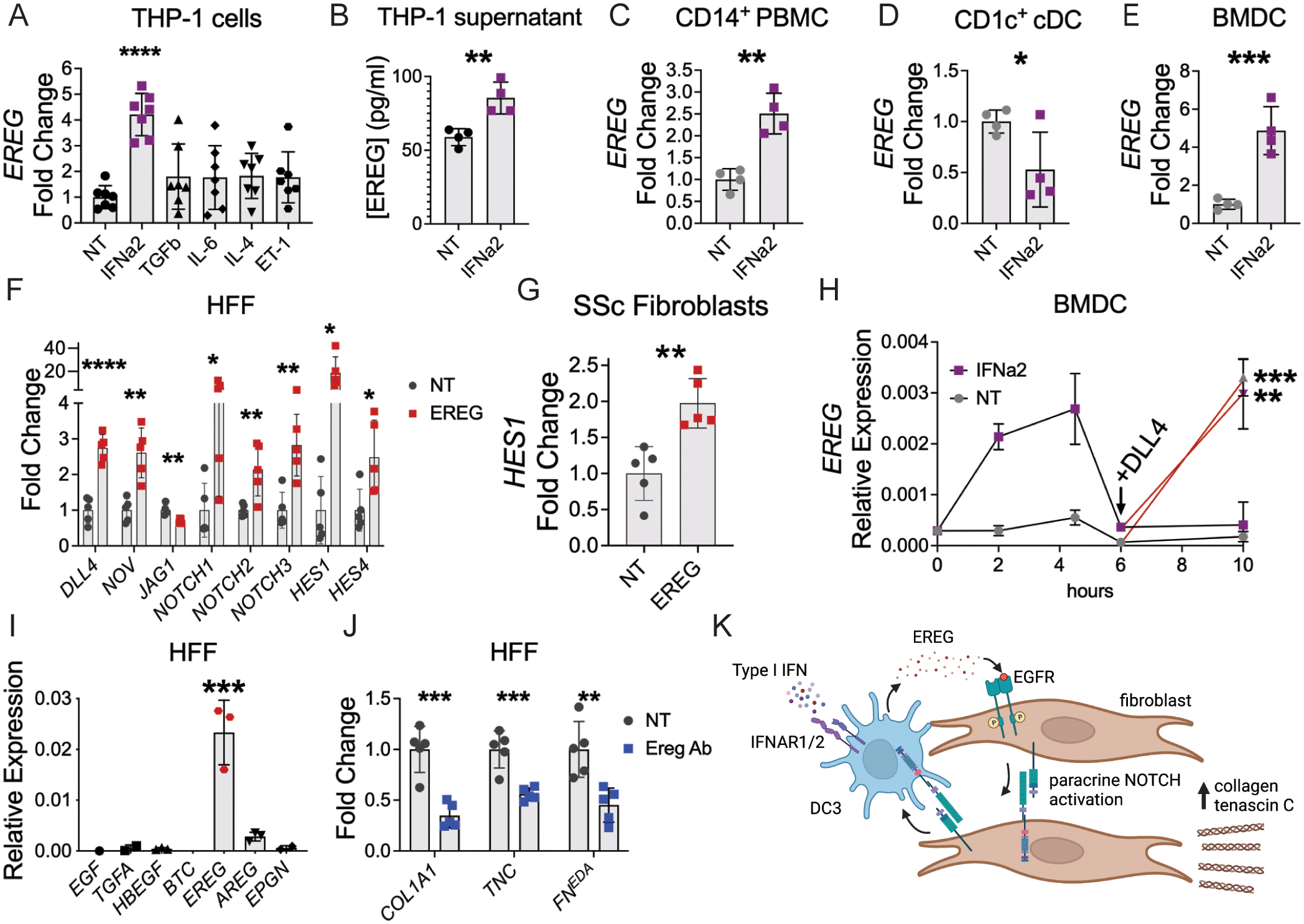
Type I interferon induces EGFR-NOTCH circuit between EREG^+^ dendritic cells and fibroblasts. (A) Expression fold change of *EREG* when THP-1 monocytes were incubated with each indicated cytokine. (B) EREG protein quantification from supernatant of THP-1 incubated with IFNa2 for 4 hours, n=4 per group, data is representative from 2 independent experiments. (C-E) *EREG* expression fold change from freshly isolated peripheral blood CD14^+^ monocytes (C) and CD1c^+^ dendritic cell precursors (D) or cultured human BMDC (E) after incubation with IFNα2. (F) Expression fold change of NOTCH ligands, receptors, and target genes by HFFs incubated with recombinant human EREG (n=5). (G) *HES1* expression fold change in SSc fibroblasts after incubation with EREG for 4 hours, n=5 per group. (H) *EREG* relative expression by BMDC primed with IFNα2 prior to exposure to NOTCH ligand DLL4 (n=3–4 per time point in each group). Statistics compare each group ± DLL4. (I) Relative expression of EGFR ligands by HFF (n=3). Genes with fewer than three points were below detectable level. (J) Changes in ECM gene expression when HFF were incubated with media alone (NT) or EREG neutralizing antibody (Ereg Ab). FN^EDA^ refers to the extra domain A-containing isoform of fibronectin (n=5 per group). (K) Model of EREG-NOTCH circuit between monocyte-derived DC3 and fibroblasts. Data are means ± SD (ns, not significant, *P < 0.05, **P < 0.01, ***P < 0.001, ****P<0.0001) analyzed with unpaired two-tailed Student t test (A-H, J) and one-way ANOVA with Tukey multiple-comparisons test (I).

Our scRNA-Seq data suggest that EREG^+^ dendritic cells are an inducible state of DC3. We therefore explored what signals regulate *EREG* expression by human immune cells. We first screened THP-1 monocytes for activating signals of *EREG* expression. Conserved regulatory elements in the first intron of the human *EREG* gene include binding sites for STAT1 complexed with STAT2 (STAT1/2), GATA3, and FOS. STAT1/2 dimerization and activation are known to occur as a result of ligand engagement of the type I interferon receptor (50). Accordingly, we observed higher expression of interferon stimulated genes in our human scRNA-Seq data (Fig. 1f). Earlier studies of *EREG* expression by cultured smooth muscle cells showed its induction by IL-6, endothelin-1, angiotensin II and α-thrombin (51, 52). We investigated *EREG* expression in response to these cytokines and found that EREG RNA and protein is induced by type I interferon IFNα2 in THP-1 monocytes (Fig. 3a, b). However, we did not observe *EREG* induction by endothelin-1, IL-6, IL-4, or TGFβ, the last two of which induce the transcription factors GATA3 and FOS, respectively.

We next investigated whether *EREG* expression could be induced by type I interferon in primary human cells. From freshly drawn human peripheral blood, we isolated CD14^+^ PBMC, which includes monocytes and DC3, and CD1c^+^ conventional dendritic cells (cDC). Whereas cDC development depends on FLT3L, DC3 development depends on GM-CSF (46). We therefore also tested human bone marrow-derived dendritic cells (BMDC), which were generated by culture of human bone marrow with GM-CSF for seven days. We incubated the different cell populations with IFNa2 for 4 hours prior to RNA isolation. We found that IFNa2 induced *EREG* expression in CD14^+^ PBMC and BMDC, but reduced its expression in CD1c^+^ cDCs (Fig. 3c–e). These data support a model in which *EREG* expression in DC3 is induced by type I interferon, which is distinct from the interferon response of cDC.

To understand how EREG regulates fibrotic pathways in dermal fibroblasts, we interrogated the expression of growth factor ligands and receptors identified in our scRNA-Seq data. In particular, we observed that NOTCH receptors were commonly expressed by immune cells and fibroblasts, suggesting a potential feedback loop. Upon incubation of confluent human foreskin fibroblasts (HFF) with recombinant EREG, we observed increased expression of NOTCH ligands *NOV* and *DLL4* along with their respective receptors *NOTCH1* and *NOTCH2* (Fig. 3f). While *NOTCH3* expression was also increased, expression of its ligand *JAG1* was reduced. Autocrine signaling by NOTCH is tightly regulated through cis-inhibition, in which ligands inhibit NOTCH receptors on the same cell (53). In EREG-treated HFFs, the expression of NOTCH ligands and receptors was accompanied by increased expression of NOTCH target genes *HES1* and *HES4*, demonstrating pathway activation in response to EREG. We confirmed that NOTCH signaling was activated by recombinant EREG in low passage SSc fibroblasts, further linking the effects of EREG to SSc pathogenesis (Fig. 3g). Thus, in dermal fibroblasts, EREG induces expression of a specific subset of NOTCH ligands and receptors, which results in activation of NOTCH pathways.

As immune cells also express NOTCH receptors, we tested whether NOTCH ligands can signal back to EREG^+^ dendritic cells. In response to IFNa2, *EREG* expression by BMDC rises, then falls back to baseline by 6 hours, suggesting a transient state of expression followed by loss of responsiveness to this cytokine (Fig. 3h). Subsequent exposure of these cells to NOTCH ligands DLL4 and NOV restores expression of *EREG* by BMDC (Fig. 3h and Fig. S4). Induction of *EREG* expression through NOTCH occurred to a similar level irrespective of interferon priming. Therefore, in addition to type I interferon, NOTCH ligands also induce *EREG* expression in primary dendritic cells and thereby act as a positive feedback signal from SSc fibroblasts to maintain EREG^+^ dendritic cells.

Given the ability of EREG to drive NOTCH activation, we hypothesized that EREG would also modulate ECM gene expression. Unlike fresh adult dermal fibroblasts, cultured HFF express *EREG* in an autocrine manner (Fig. 3i). We took advantage of this observation to test if an EREG neutralizing antibody (Ereg Ab) could reduce ECM gene expression in cultured HFF, and found that it significantly reduced expression of *COL1A1* as well as the fibrotic ECM genes tenascin C (*TNC*) (54) and the extra domain A-containing isoform of fibronectin (*FN*^*EDA*^) (55) (Fig. 3j). Overall, these results reveal a cellular circuit (Fig. 3k), in which type I interferon induces *EREG* expression in DC3, EREG^+^ dendritic cells in turn activate NOTCH signaling in fibroblasts, and fibroblast-derived NOTCH ligands bind NOTCH receptors on EREG^+^ dendritic cells to maintain *EREG* expression. Furthermore, EREG-mediated EGFR activation is necessary for expression of multiple fibrotic ECM genes.

### *Ereg* has defined expression patterns in mouse skin and lung fibrosis

**Fig. 4.**
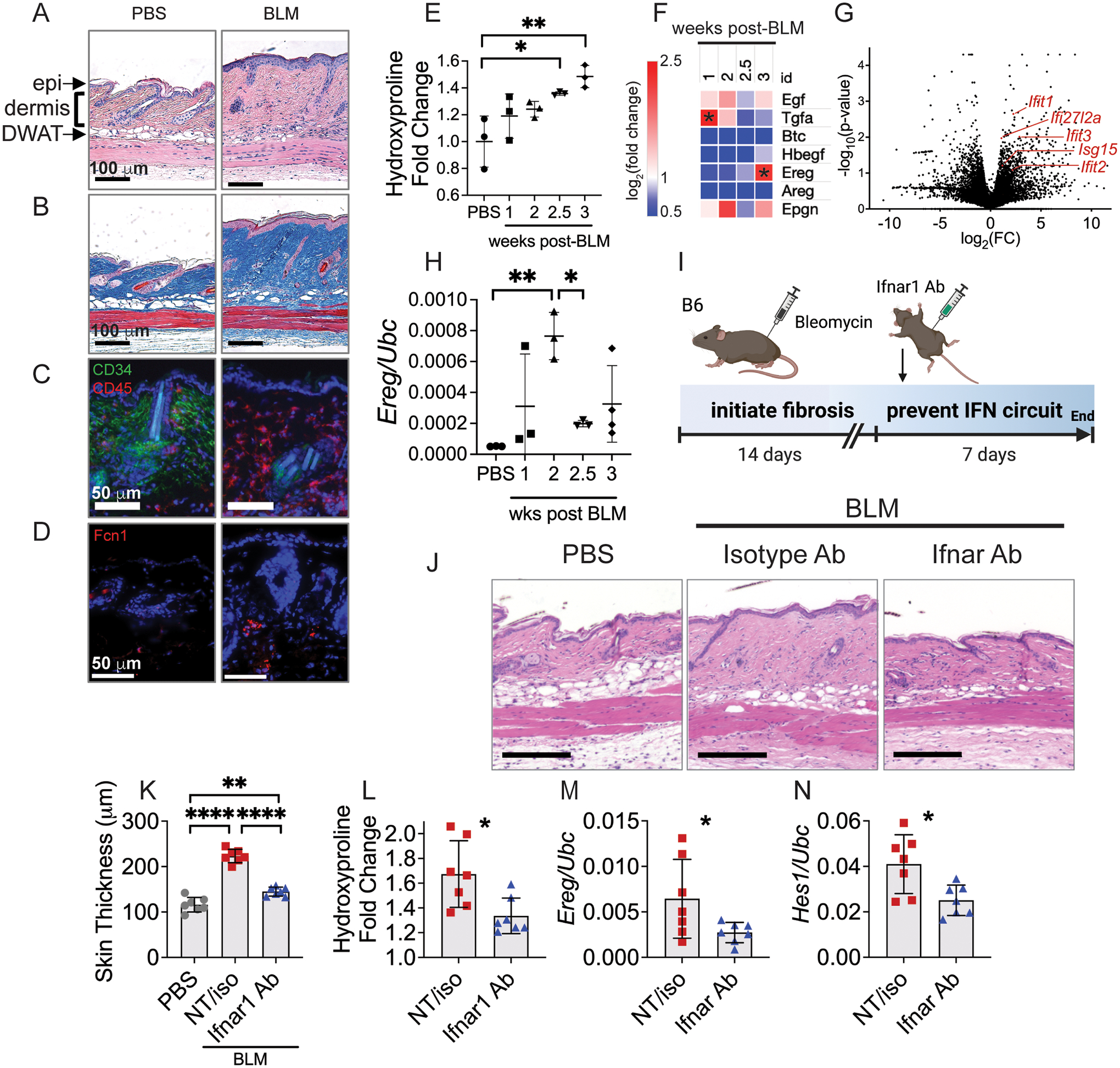
*Ereg* has defined expression patterns during mouse skin and lung fibrosis. (A-C) B6 mice were injected subcutaneously with 0.2 mg bleomycin (BLM) and 3 weeks later skin was stained for hematoxylin and eosin (A) and trichrome (B). Epidermis (epi), dermis (dermis) and dermal white adipose tissue (DWAT) are highlighted on histology. (C, D) Immunofluorescence images of PBS and BLM-treated skin 3 weeks post-injection. (E) Hydroxyproline content of the skin at different time points after subcutaneous bleomycin injection (n=3 per group). (F) Heatmap of mean log_2_(expression fold change) of ECM genes and EGFR ligands at different time points after subcutaneous bleomycin injection compared to the mean of each group and PBS controls, n=3 per time point. (G) Bulk RNA sequencing of dendritic cells isolated from fibrotic skin of Mgl2^DTReGFPpANeo^ mice 3 weeks after subcutaneous bleomycin injection compared to PBS controls (n=3 per group). (H) Relative expression of *Ereg* at different time points after intratracheal bleomycin administration to B6 mice. (I) B6 mice were injected with bleomycin subcutaneously, then at 2 weeks injected intraperitoneally with Ifnar1-blocking antibody (Ifnar1 Ab), isotype control antibody (iso) or not treated (NT). No significant differences were found between NT and isotype Ab control groups, so they were combined for clarity. At 3 weeks, skin was analyzed for histology (J), dermal skin thickness (K), hydroxyproline (L), and gene expression (M, N). Data from E and F, G, and H are single independent experiments. Data in J-N are aggregated from two separate experiments. Data are means ± SD (ns, not significant, *P < 0.05, **P < 0.01, ***P < 0.001, ****P<0.0001) analyzed with one-way ANOVA with Tukey multiple-comparisons test (E, F, H, K) and unpaired two-tailed Student t test (L-N).

Different mouse models of SSc recapitulate characteristics of distinct SSc disease subsets (56). To best interrogate the role of EREG in SSc *in vivo*, we required a mouse model that depends on myeloid APC and induces both skin and lung fibrosis. The bleomycin model, by either subcutaneous or intratracheal injection, achieves each of these requirements (57, 58). We tested multiple dosing protocols of bleomycin (modified from Yamamoto et al. (59)) and found that in our hands, a single subcutaneous dose of bleomycin 0.2 mg per mouse (6- to 10-week-old B6 females) induced skin changes by 3 weeks post-injection that were reminiscent of human SSc. Histologic changes included dermal thickening, loss of dermal white adipose tissue (DWAT), and loss of CD34^+^ cells (60) (Fig. 4a–c). Similar to human SSc skin, DC3 were apparent at the border of mouse fibrotic dermis as marked by Fcn1 (Fig. 4d). By 2.5 weeks after bleomycin injection, there was significantly increased dermal collagen by measurement of hydroxyproline (Fig. 4e). These findings are consistent with prior literature showing that B6 mice develop fibrosis by 3 weeks (58) and that a single dose of bleomycin can model pulmonary fibrosis (61). For lung fibrosis, we administered bleomycin sulfate (0.016 mg) intratracheally as a single dose as previously described (62).

To characterize the dynamics of EGFR ligand expression in bleomycin-induced skin and lung fibrosis, we performed a time course of gene expression in each tissue. One week after bleomycin injection into the skin, we observed elevated expression of the high-affinity EGFR ligand transforming growth factor alpha (*Tgfa*) in fibrotic compared to PBS-treated control skin (Fig. 4f). This aligns with previous reports that *Tgfa*-deficient mice are protected from developing lung fibrosis (63). Three weeks after bleomycin injection, we observed increased *Ereg* expression, which coincided temporally with elevated expression of interferon signature genes by dendritic cells (Fig. 4g). These observations suggest that the initial development of fibrosis is influenced by Tgfa, but interferon-activated EREG^+^ dendritic cells are likely important later in the chronic phase, represented by three weeks in mice. We similarly measured the expression of *Ereg* in mouse lungs after intratracheal administration of bleomycin and found that it was elevated 1–2 weeks after bleomycin exposure (Fig. 4h). Therefore, in mice exposed to bleomycin, *Ereg* has defined expression patterns during both skin and lung fibrosis, rendering the model useful for understanding EREG-EGFR signaling in relation to human disease.

Expression of *EREG* by DC3 in vitro was regulated by exposure to type I interferon. To test whether EREG-EGFR-NOTCH signaling depends on interferon in vivo, we blocked interferon activation in the bleomycin skin fibrosis model using a neutralizing antibody. Two weeks after subcutaneous bleomycin injection, mice were treated intraperitoneally with an interferon receptor blocking antibody (Ifnar1 Ab, Fig. 4i), which we hypothesized would prevent *Ereg* expression by DC3 and potentially improve fibrosis. Indeed, compared to vehicle and isotype antibody-treated controls, Ifnar1 antibody-treated mice showed reduced skin thickness and collagen content (Fig. 4j–l). Furthermore, relative expression of *Ereg* and the Notch target gene *Hes1* were reduced in the Ifnar1-antibody treated mice (Fig. 4m, n). This shows that when administered prior to *Ereg* induction, interferon inhibition removed the stimulus for *Ereg* expression, and thereby prevented EGFR-mediated NOTCH activation. Thus, *Ereg* expression in skin fibrosis is interferon-dependent in vivo and interferon blockade can improve fibrosis when administered during the appropriate window of time prior to EREG activation of EGFR and downstream NOTCH signaling.

### EREG inhibition alleviates murine skin and lung fibrosis

**Fig. 5.**
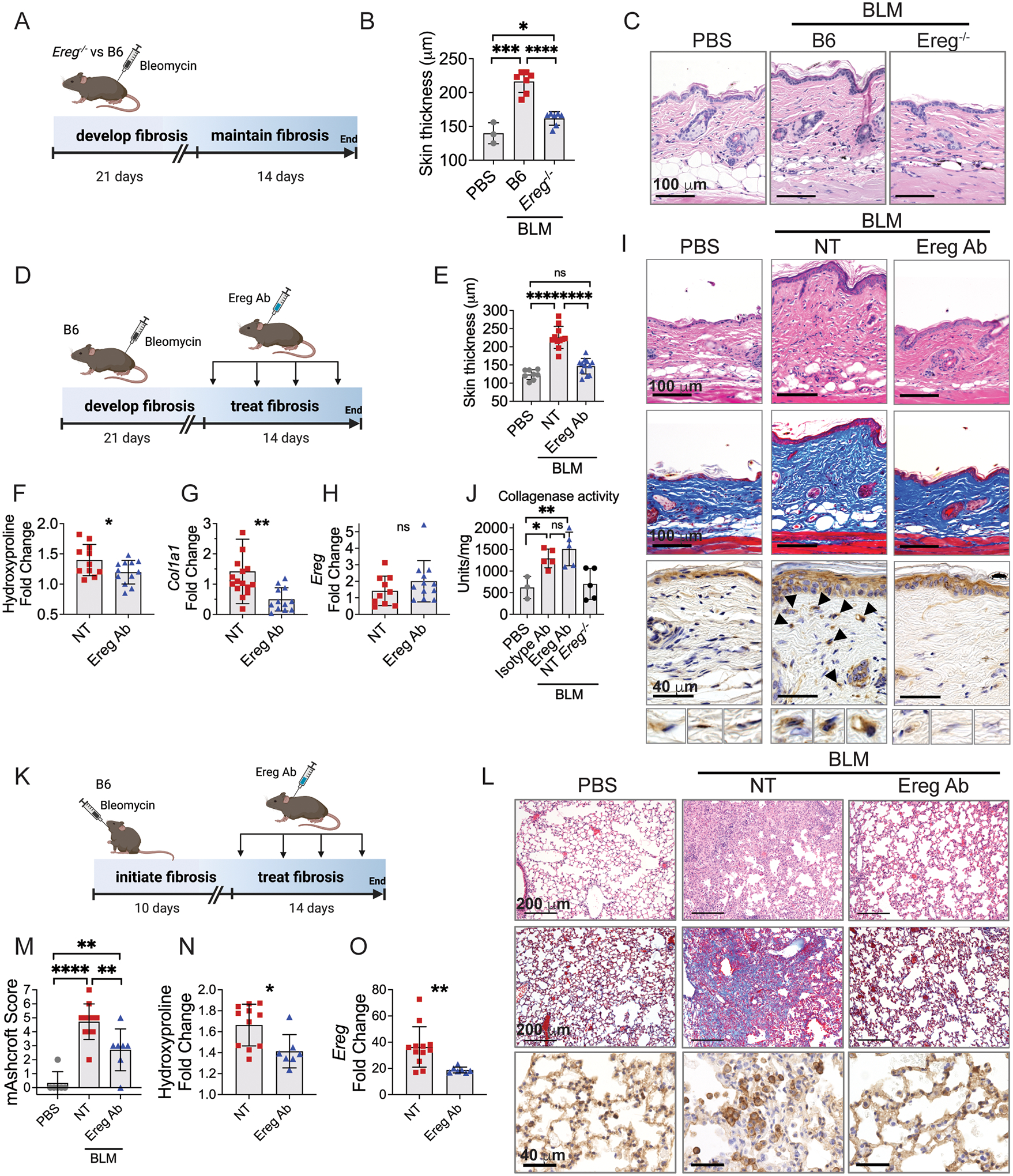
EREG inhibition alleviates mouse skin and lung fibrosis. (A-C) Diagramed in (A), cohorts of B6 and *Ereg*^−/−^ mice were injected with bleomycin subcutaneously and 35 days later analyzed for skin thickness (B) and histology (C). (D-I) Diagramed in (D), 21 days after bleomycin injection mice began treatment with Ereg antibody (Ereg Ab) compared to controls treated with PBS (NT) for two weeks. Skin was analyzed for dermal thickness (E), hydroxyproline (F), gene expression (G, H) and histology (I), with H&E staining on the top row, trichrome in the middle row, and pEGFR immunohistochemistry (IHC) on the bottom row, n=8 (PBS), 11 (BLM), and 12 (Ereg Ab). (J) B6 and *Ereg*^−/−^ mice were injected with bleomycin or PBS and 3 weeks later B6 mice were treated for 1 week with Ereg Ab or isotype control Ab, n=3 (PBS), 5 (isotype Ab), 5 (Ereg Ab), and 5 NT *Ereg*^−/−^. (K-L) As diagramed in (K), 10 days after intratracheal bleomycin mice were treated with Ereg Ab for two weeks. Lungs were analyzed for histology (L), modified Ashcroft score (M), hydroxyproline (N) and *Ereg* gene expression (O), n=6 (PBS), 11 (BLM), and 7 (Ereg Ab). Histology images of skin and lung are 10x and 20x magnification, respectively. IHC images are 40x magnification. Data are means ± SD (ns, not significant, *P < 0.05, **P < 0.01, ***P<0.001, ****P<0.0001) analyzed with unpaired two-tailed Student t test (F-H, N, O) and one-way ANOVA with Tukey multiple-comparisons test (B, E, J, M). Data for J is single experiment and data for A-C, D-I, and K-O are aggregated from two independent experiments.

Because elevated *Ereg* expression in fibrotic murine skin was delayed until 3 weeks post-bleomycin, we hypothesized that it would be most important during the chronic phase and dispensable for the development of fibrosis. It takes B6 mice 3 weeks to develop skin fibrosis in response to bleomycin (58). Therefore, to assess at what time EREG is required for development versus maintenance of skin fibrosis, we examined the response of *Ereg*-deficient mice to bleomycin 3 and 5 weeks after exposure. At 5 weeks after bleomycin injection, *Ereg*^−/−^ mice showed decreased dermal skin thickness compared to wild type mice, suggesting that EREG supports the persistence of fibrosis (Fig. 5a–c). Decreased skin fibrosis in *Ereg*^−/−^ mice was not due to any defect in fibrosis development because at 3 weeks, *Ereg*^−/−^ mice showed dermal thickening and elevated collagen similar to their wild type counterparts (Fig. S5a–c). Together, these findings show that EREG is required for the persistence of fibrosis as opposed to fibrosis establishment.

To specifically assess the temporal role of EREG in the bleomycin model of skin fibrosis, we inhibited EREG by twice weekly subcutaneous injection of a neutralizing antibody starting 3 weeks after bleomycin injection (corresponding to its peak of expression) (Fig. 5d). The antibody was injected at a distant location (dorsal neck) from the bleomycin (lower back). In wild type mice, two weeks of treatment with an EREG antibody (Ereg Ab) resulted in complete normalization of dermal skin thickness, 50% reduction in collagen protein, and 190% reduction in *Col1a1* expression to below PBS control levels (Fig. 5e–g). Ereg Ab treatment was also associated with decreased pEGFR staining in dermal cells, supporting its inhibition of EGFR activation by EREG^+^ dendritic cells (Fig. 5i). The Ereg Ab did not alter expression of *Ereg* in the skin, suggesting it does not deplete EREG^+^ dendritic cells (Fig. 5h) and treatment with an isotype antibody did not show a similar benefit in this model compared to non-treated controls (Fig. S5d–i). Thus, inhibition of EREG reverses fibrosis in the skin by decreasing collagen expression and accumulation.

Collagen degradation is a complex activity which occurs by extracellular proteases and intracellular catabolism by fibroblasts and macrophages. To test whether extracellular collagen degradation is accelerated by Ereg Ab in the bleomycin model, we used a fluorescent collagen degradation assay of mouse skin after 1 week of treatment with Ereg Ab compared to isotype control as well as PBS and non-treated *Ereg*^−/−^ controls. We found that mice exposed to either antibody therapy showed an approximately 3-fold greater rate of collagen degradation compared to PBS and *Ereg*^−/−^ controls (Fig. 5j). These results suggest that reduction in collagen expression and increased collagen degradation by EREG inhibition led to reduced collagen in the skin in a 2-week time frame of these mouse model studies.

Based on the efficacy of EREG inhibition to reverse skin fibrosis, we examined if EREG inhibition could also treat lung fibrosis. *Ereg* expression in lungs increased 1–2 weeks after intratracheal bleomycin exposure (Fig. 4h), so we started 2 weeks of antibody treatment on day 10 after bleomycin administration (Fig. 5k). In these mice, treatment with subcutaneous Ereg Ab prevented the development of large fibrotic masses and preserved visible alveolar septae (Fig. 5l). These findings corresponded with significant improvement in lung fibrosis measures with a 2-point reduction in modified Ashcroft score and 38% reduction in collagen protein (Fig. 5m, n). Ereg Ab treatment resulted in loss of pEGFR^+^ cell clusters in fibrotic areas of lung compared to the non-treated group (Fig. 5l), supporting the model of EREG-dependent EGFR activation. Ereg Ab treatment also decreased the expression of *Ereg* from whole tissue, albeit not to the level of PBS controls, which reflects lower expression of *Ereg* by EREG^+^ dendritic cells or fewer number of EREG^+^ dendritic cells in the Ereg Ab treated fibrotic lungs (Fig. 5o). Altogether, these findings show that EREG inhibition attenuates skin and lung fibrosis in mice.

### EREG inhibition reverses human skin and lung fibrosis

**Fig. 6.**
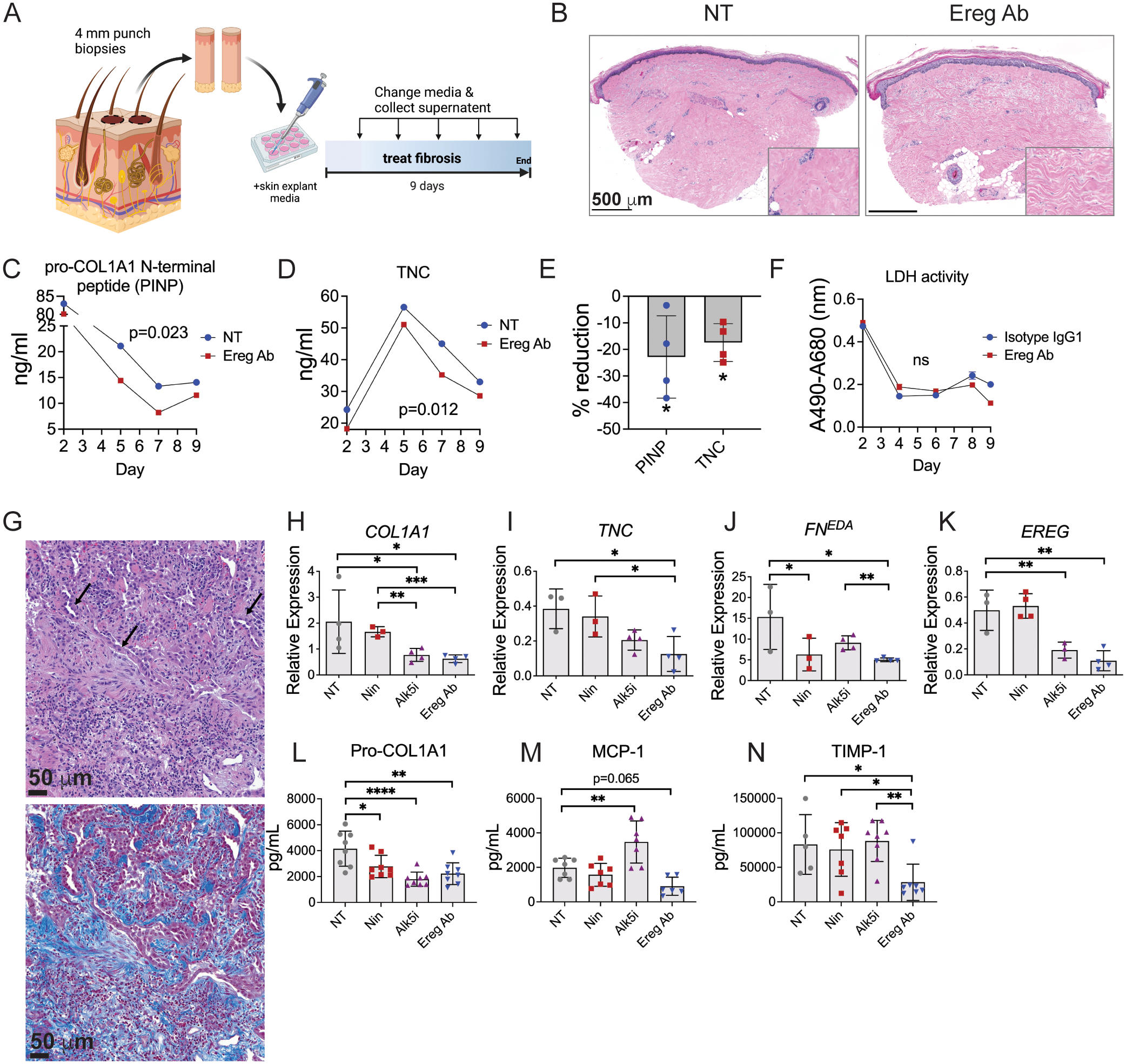
EREG inhibition reverses human skin and lung fibrosis. (A) Experimental diagram depicting adjacent punch biopsies obtained from the forearm of a patient with diffuse cutaneous SSc, which were cultured for 9 days in media alone (NT) or with addition of EREG neutralizing antibody (Ereg Ab). (B) Histology of cultured skin explants, with inset showing higher magnification of dermal collagen. (C, D) Skin explant media was analyzed for pro-COL1A1 N-terminal peptide (PINP) and TNC. (E) Percent reduction of protein by Ereg Ab treatment compared to NT control. (F) LDH activity of skin explant supernatants from patient SSc7. (G-N) Fresh explanted lung tissue from a deceased patient donor with familial idiopathic pulmonary fibrosis was processed for histologic staining, which showed fibroblastic foci formation and hyperplasia of alveolar type II epithelial cells, indicated by arrows (left panel H&E, right panel trichrome). The same tissue was cut into cubes and cultured for 10 days in the presence of the multikinase inhibitor nintedanib (Nin), the Alk5 inhibitor A-1544033 (IN-1130) (Alk5i), EREG antibody (Ereg Ab) or non-treated vehicle control (NT). (H-K) Relative expression of indicated genes, n=4 per group. (L-N) Protein secretion of indicated genes measured by ELISA, n=8 per group. ELISA samples with poor signal and qPCR outliers identified by Grubbs’s test with alpha = 0.05 were excluded. Data are means ± SD (ns, not significant, *P < 0.05, **P < 0.01, ****P < 0.0001) analyzed with paired two-tailed Student t test (C-E) comparing NT and Ereg Ab treated samples. In (H-N), comparison of each inhibitor to NT control was analyzed by one-way ANOVA with Dunnett’s multiple-comparisons test whereas Ereg Ab was individually compared to Nin and Alk5i by unpaired two-tailed Student t test.

To investigate whether EREG inhibition could be translated to treat human SSc patients, we tested EREG neutralization on skin explants obtained from a patient with worsening diffuse cutaneous SSc. At the time of biopsy, the patient had a mRSS of 45, positive RNA polymerase III antibody, and previously failed treatment with mycophenolate mofetil. Adjacent 4 mm punch biopsies from the right forearm were obtained and cultured for 9 days in the presence or absence of Ereg neutralizing antibody (Fig. 6a). We found that EREG inhibition resulted in improved histologic appearance of the skin with reduced collagen fiber thickness (Fig. 6b). Measurement of COL1A1 and TNC from the skin explant supernatant showed significantly reduced production of each ECM gene by SSc skin treated with Ereg Ab compared to NT control (Fig. 6c–e). To test whether the effects we observed were due to cell death within skin explants, we obtained skin biopsies from another patient with SSc (SSc7) whose skin disease was improving on therapy. We found no difference in LDH activity of explant supernatant between EREG antibody and isotype control treated samples (Fig. 6f). LDH levels were higher on day 2, then decreased for the remainder of the 9-day culture, supporting an overall low level of cell death. Thus, EREG inhibition improved key markers of skin fibrosis by skin explants from a patient with severe treatment refractory SSc.

To interrogate the effects of EREG inhibition on human lung fibrosis, we investigated the impact of EREG treatment on lung explants from a patient with IPF. Pathologic assessment confirmed the diagnosis of early IPF, as evidenced by the presence of fibroblastic foci and hyperplasia of alveolar type II epithelial cells (Fig. 6g). The impact of EREG antibody was compared to the multikinase inhibitor nintedanib and a TGF-βRI/Alk5 small molecule inhibitor. Ereg Ab reduced expression of *COL1A1*, *TNC*, and *FN*^*EDA*^ each by approximately 70% (Fig. 6h–k). Ereg Ab and TGF-βRI/Alk5 inhibition were similarly more effective than nintedanib in reducing *COL1A1* gene expression (Fig. 6h). Ereg Ab showed superior reduction in *TNC* and *FN*^*EDA*^ expression compared to nintedanib and TGF-βRI/Alk5 inhibitor, respectively (Fig. 6i, j). Both Ereg Ab and TGF-βRI/Alk5 inhibitor reduced *EREG* expression similar to what we observed in mouse lung fibrosis, suggesting a regulatory link between *EREG* expression and TGFβ signaling (Fig. 6k). On the protein level, we observed significant reduction in the levels of pro-COL1A1 by all three inhibitors (Fig. 6l). Anti-EREG treatment also significantly reduced TIMP-1 levels, which were unaffected by nintedanib and Alk5i, and trended toward significance in its reduction of MCP-1 (CCL2) (Fig. 6m, n). These results highlight a superior ability of EREG-blockade to impact ECM-remodeling in IPF tissue compared to TGFβ inhibition and the existing FDA-approved antifibrotic treatment nintedanib. Overall, our results support the potential for anti-EREG treatment as an antifibrotic treatment in IPF and by extension, SSc-associated pulmonary fibrosis.

In summary, through scRNA-Seq analysis of SSc skin and lung, we identify EREG as a pathogenic EGFR ligand produced by DC3 dendritic cells and whose expression is regulated by type I interferon. Direct inhibition of EREG reverses fibrosis in both mouse skin and lung models and patient explants, identifying it as a potential therapy for patients suffering from SSc and other fibrotic diseases by targeting a fundamental immune-fibroblast interaction.

## Discussion

In this study, we reveal how *EREG* expression by DC3 dendritic cells is a crucial signal to maintain fibrosis in the skin and lungs. Multiple approaches including scRNA-Seq from patients, mouse models, and patient explants demonstrated the importance of EREG-EGFR signaling in SSc and other fibrotic diseases. We show that *EREG* expression is induced by type I interferon and co-expressed with alarmins *S100A8* AND *S100A9*, which aligns our findings to previous clinical studies that associated circulating levels of type I interferon and *S100A8/A9* with severity of SSc skin and lung fibrosis (35, 48, 64). Recently, *FCN1* and *EREG*-expressing DC were found to be expanded in more severe SSc skin disease (65). We further tested if *EREG* itself was associated with extent of SSc skin disease using a recently published large-scale dataset (49). An important caveat to scRNA-Seq studies is the effect of tissue digestion on gene expression, in particular induction of immediate early and tissue repair genes (66). Despite longer digest time used by Gur et al. (49), we found *EREG* expression to be significantly associated with mRSS. Thus, expression of *EREG* by DC3 correlates across multiple independent studies with the presence of SSc and its severity.

Earlier studies showed that global EGFR inhibition could prevent the development of skin, liver, and kidney fibrosis (8–10). Taken alone, these observations suggested that EGFR inhibitors could be therapeutically effective in patients. However, clinical studies of broadly acting tyrosine kinase inhibitors for treatment of skin and lung fibrosis have been marred by the development of serious adverse events (67, 68) and EGFR inhibition can paradoxically induce pulmonary fibrosis in patients (69, 70) and mice (11). Independent of its kinase activity, EGFR promotes cell survival as a protein scaffold, by which it stabilizes multiple other key survival proteins (see e.g. (71)). Small molecule tyrosine kinase inhibitors (TKI) gefitinib and erlotinib, which were used in the mouse fibrosis studies, inhibit the ATP binding pocket of the active conformation of EGFR. Kinase inhibition in this manner induces EGFR quasi-dimerization (72–74), in which the function of EGFR is shifted to its kinase-independent activity. We hypothesize that in addition to its canonical kinase signaling activity, EGFR kinase-independent signaling is important in fibrosis pathogenesis. Thus, TKI such as gefitinib and erlotinib may have variable effects on fibrosis depending on contributions of EGFR kinase and scaffolding functions in the tissue of interest. Specifically, in EGFR kinase-driven fibrosis, TKI should be protective, but TKI may exacerbate fibrosis when EGFR scaffolding is important. Therefore, targeting the upstream EGFR ligand EREG should avoid the risk of fibrosis exacerbation associated with TKI therapies.

EREG is one of seven cell-surface EGFR ligands, previously reported to protect the gastrointestinal tract from dextran sulfate sodium colitis (75) and to signal with BTC and AREG to induce maturation of the ovarian follicle (76). EREG-EGFR activation is one of multiple important signaling processes that are active in fibrosis, including TGFβ, IL-1 and type 2 family cytokines (77). However, none of these cytokines are entirely specific to fibrosis. Rather, the cytokine milieu in fibrotic tissue likely determines disease phenotype. Accordingly, *EREG*-expressing DC3 have also been reported to be increased in psoriasis skin (78), which suggests targeting EREG may be therapeutic in other inflammatory skin conditions. More generally, our findings closely relate to the concept that developmental programs are co-opted in inflammatory skin disease (79). In the context of fibrosis, we show that EGFR is one developmental program that is aberrantly activated, and how targeting EREG as its pathogenic ligand demonstrates that inhibition of upstream signals have translational potential as a therapeutic strategy.

Fibrosis occurs in multiple phases, including initial development followed by maintenance. Molecular signals required to maintain fibrosis are different from those that drive development. This distinction is exemplified in prior work demonstrating the importance of TLR4 activating signals to drive fibrosis persistence (80). Our murine studies using bleomycin-induced skin and lung fibrosis indicating that *Ereg* expression is elevated during persistent disease (post-development) should be viewed in the same manner. Additionally, better efficacy by the EREG antibody to improve skin compared to lung fibrosis may reflect tissue-specific differences in maintenance signals. A conceptually similar phenomenon of development vs maintenance may occur in long-term tissue remodeling and scar formation after myocardial infarction injury, in which lineage-traced myofibroblasts are retained in the scar but lose proliferative capacity and αSMA expression over time (81). Future studies that address the importance of time and natural history of disease will be needed to better understand how fibrosis occurs as a dynamic process.

Human skin explant models and skin equivalents are a highly active area of research to mimic human illnesses ex vivo. Human skin explants induced to become fibrotic are viable for up to 14 days (80) and can be cultured for pharmacodynamic therapy up to 9 days (82). We modified these protocols to investigate the effects of EREG inhibition on SSc skin and IPF lung. A limitation of our study is the low number of explant samples, which is due their high value but limited availability because we directly used human tissue of each disease. Also, in order to control for disease subtype, we enrolled patients with active diffuse SSc, but did not exclude those with longer disease duration or those on therapy. In explants from our patient with severe SSc who previously failed therapy with mycophenolate mofetil (MMF), EREG inhibition improved skin fibrosis by histology and ECM protein measurements. The primary mechanism of action of MMF occurs by impairing nucleotide synthesis of T and B lymphocytes, and SSc patients with higher lymphoid gene expression modules are more responsive to MMF therapy, but not SSc patients with myeloid signatures (83). Our patient’s skin may have improved in response to EREG inhibition in contrast to prior clinical treatment with MMF because EREG is specifically a myeloid-derived signal. The resulting combination of reduced collagen expression with increased collagen turnover mediated by the EREG antibody mechanistically explains the improvement in skin and lung collagen and other ECM modifying genes. Together these results highlight how gene expression signatures may be useful to select targeted therapies for patients in the clinic and that EREG inhibition may be a promising strategy to target SSc and other fibrotic diseases.

## Materials and Methods

### Study design

This study aimed to identify cellular signals that drive skin fibrosis using scRNA-Seq of skin samples obtained from 5 patients with diffuse SSc and 6 healthy control volunteers. Data were analyzed by differential gene expression and ligand-enrichment analysis, in which we discovered enriched EREG-EGFR interaction. EREG-EGFR enrichment was reproducible across five publicly available SSc skin and lung scRNA-Seq datasets. We characterized EREG and EGFR activation in tissue using immunohistochemistry and immunofluorescence of human skin and lung tissue. Mechanistic studies of EREG regulatory signals were performed in vitro with human immune cells and fibroblasts. In vivo validation of EREG signaling during persistence of fibrosis was assessed with EREG knockout mice and a neutralizing antibody in the bleomycin mouse model of skin and lung fibrosis, as well as human skin and lung explants.

### Study Patients

Skin biopsies from five patients with diffuse cutaneous SSc and five healthy controls were analyzed with single-cell RNA sequencing. Adult patients with diffuse scleroderma (systemic sclerosis) diagnosed by American College of Rheumatology criteria (84) and healthy controls were recruited for study approved by the Yale Human Investigation Committee (HIC# 1511016816). Patients with active disease were enrolled and not excluded based on number of years since diagnosis and concomitant therapy. The clinical diagnoses of scleroderma was confirmed by histopathology of the skin in all patients. Women and minorities were not excluded from this study based on sex/gender, race, or ethnicity. The patient’s clinical data, including age, sex, age of onset of disease, duration of disease, family history of autoimmune disease, and current and previous treatments, were reviewed by Dr. Odell. Dr. Odell was the only member with access to de-identified patient data. Exclusion criteria included evidence of overlapping autoimmune disease, chronic bloodborne infections including HIV and hepatitis B and C, and inability to provide informed consent. Healthy controls were excluded if they had personal or family history of autoimmune disease. Therapy of patients on immune modifying medications was not interrupted for the study and biopsies of those on ECP was performed just prior to their treatment to maximize time since prior treatment.

### Single-cell tissue preparation and RNA sequencing

After anesthetizing a 1–2cm area of skin with 1% lidocaine hydrochloride with epinephrine 1:100,000, two adjacent punch biopsies measuring 6 mm and 3 mm were performed. The 3 mm biopsy was fixed in 10% neutral buffered formalin and the 6 mm biopsy was immediately processed for single cell RNA library preparation. The entire 6 mm specimen was first incubated in RPMI 1640 medium (Gibco) containing 5% fetal bovine serum (5% FBS/RPMI) and 10 mg/ml Dispase II (Sigma D4693–1G) for 45 minutes at 37° C shaking at 200–250 rpm. The 6 mm sample was then removed from the media and minced with sterile iris scissors, followed by digestion with Liberase TM (Sigma) 0.5 mg/ml and DNase I 30 Units/ml in 5% FBS/RPMI for 45 minutes at 37° C shaking at 200–250 rpm. The resulting single cell suspension was then filtered through a 70 μm nylon membrane and washed. Live cells were sorted on a FACSAria at the Yale Flow Core and their final concentration and viability quantified with Trypan blue on a hemacytomer. The cells were pelleted and suspended in phosphate buffered saline containing 0.04% bovine serum albumin between 500–1000 cells/μl. 3000–6000 cells with greater than 80% viability were submitted to the Yale DNA Sequencing facility for generation of single-cell cDNA libraries using the Chromium Single Cell Controller (10x Genomics). For the skin digestion time course of Ereg expression, a healthy 6-week-old female B6 mouse was euthanized and 2 mm punch biopsies from the back (2 per sample, 3 samples per time point) were digested with Liberase TM and DNase I in 5% FBS/RPMI as described above. After digest, cells were pelleted and processed for qPCR as described below.

### Single-cell analysis

Each cDNA library generated from a 6 mm skin sample was sequenced paired-end on 1 lane with 75 base-pair read length using the Illumina HiSeq 2500 System generating at least 75,000 reads per cell. The 10x genomics Cell Ranger pipeline was then used to align the reads, perform clustering and gene expression analysis, and aggregate the samples with normalized read counts. t-Distributed Stochastic Neighbor Embedding (tSNE) plots used 10 principle components. The raw matrices from Cell Ranger were also processed with Seurat version 3 R toolkit for single cell genomics (85, 86) to filter low quality samples, followed by data normalization (log normalized using the default scaling factor of 10000), scaling, PCA analysis, UMAP clustering, and generation of violin plots, and subsequently analyzed with CellphoneDB v2.0 (39, 40) for receptor-ligand enrichment. All 10x experiments were completed with the same 3’ chemistry and high-throughput sequencer to avoid batch effects. Raw matrices from skin and lung scRNA-Seq were downloaded from the NCBI Gene Expression Omnibus (GSE138669 (15), GSE122960 (41), GSE128169 (42), GSE132771 (33), and GSE195452 (49)) and analyzed as above using the Seurat toolkit followed by CellphoneDB. To best match the age and sex of the SSc samples in Reyfman et al (41), donor numbers 1, 3, 4, and 7 were used for healthy control data. Heatmaps of gene expression were generated from the cell clusters in the 10x Loupe Browser v5 with MORPHEUS software (https://software.broadinstitute.org/morpheus).

### Immunohistochemistry of skin and lung sections

From patient skin, 3 mm skin biopsy samples were fixed in 10% neutral buffered formalin for 24 hours prior to embedding in paraffin. Samples were processed at the Yale Pathology Tissue Services. For immunohistochemistry analysis, 5 μm sections were cut and slides were deparaffinized and rehydrated to distilled water. They were then placed in TBS with tween. This is the same solution that is used in subsequent washing steps. Endogenous peroxidase was blocked using 3% hydrogen peroxide and then rinsed. The slides were then treated with Proteinase K for 7 min and rinsed. For pEGFR and vimentin co-labelling, heat-induced epitope retrieval was utilized. The slides were incubated with primary antibody, rinsed and the antibodies detected with HRP-conjugated secondary antibody. DAB was used to identify the reaction, then the slides were washed and counterstained in hematoxylin, dehydrated, cleared and mounted with resinous mounting media. Quantification of EREG and pEGFR positive cells was completed in blinded fashion by scoring the number of positive cells in 10 high powered fields of one slide each of skin samples from study patients SSc1, 3, and 4.

### Immunofluorescence of skin sections

For immunohistochemistry analysis, a strip of skin was placed in OCT, frozen and stored at −80° C. Using Leica CM1850 cryostat, 8–10 μm sections were cut and used immediately or stored at −20° C. After briefly drying tissue sections at room temperature, they were sequentially fixed in 4% paraformaldehyde, permeabilized with 0.1% Triton X-100, blocked with serum-free protein block (Dako), and treated with TrueBlack Lipofuscin Autofluorescence Quencher (Biotium). Fc receptors were blocked with Human TruStain FcX (Biolegend) or anti-mouse CD16/32 antibody (Biolegend). Sections were then incubated with the indicated primary antibodies for 1 hour at room temperature or overnight at 4° C, then labelled with the corresponding secondary antibodies. Slides were imaged with a Keyence BZ-X800 microscope. A table of primary antibodies is included in Table S2.

### Animals

Wild type C57BL/6 were purchased from Charles River Laboratories. Mgl2^DTReGFPpANeo^ (Mgl2-DTR-GFP) mice were kindly provided by Akiko Iwasaki (Yale University). All mice were maintained at the Yale University School of Medicine Animal Resources Center. Mouse experiments were conducted on 6- to 10-week-old females under a protocol approved by the Yale University Institutional Animal Care and Use Committee and in accordance with AAALAC guidelines.

### Bleomycin mouse models of fibrosis

Mice were anesthetized using an isoflurane precision vaporizer. To induce skin fibrosis, mice were laid on their abdomen, then a 2×2 cm area of fur removed with electric clippers, and then injected subcutaneously with 0.2 mg bleomycin sulfate (Sigma B8416) diluted in 0.2 ml sterile PBS (10 mg/kg) or 0.2 ml PBS vehicle control using 30-gauge needle. To induce lung fibrosis, mice were suspended vertically by their incisors and administered bleomycin sulfate 1.25 U/kg intratracheally in 60 μL PBS as previously described (62). From lung specimens, the right three lobes were used for hydroxyproline quantification, the left upper lobe for histology, and the left lower lobe for gene expression. Modified Ashcroft score (87) of the lung was calculated in a blinded manner. All experiments have wild type (B6) controls to account for variation in potency of bleomycin lots to induce fibrosis. Fibrosis was measured on day 21 after bleomycin injection unless otherwise noted. EREG neutralizing antibody (R&D Systems MAB1068 clone 189611) or mouse IgG2a isotype control (Bio X Cell BE0085) 10 mg/kg diluted in 100 μl PBS was given subcutaneously twice weekly on the dorsal neck of anesthetized mice. To block type I interferon signaling, 1.67 mg IFNAR-1 antibody (Bio X Cell clone MAR1–5A3) diluted in 0.5 mL PBS was administered intraperitoneally as a single dose 2 weeks after bleomycin injection. For bulk RNA sequencing of skin dendritic cells, Mgl2^DTReGFPpANeo^ mice were injected subcutaneously with bleomycin as above, but not injected with diphtheria toxin. 3 weeks later, the affected skin was harvested and digested with Liberase TM (Sigma) 0.5 mg/ml and DNase I 30 Units/ml in 5% FBS/RPMI for 1 hour at 37° C shaking at 200–250 rpm. The resulting single cell suspension was then filtered through a 70 μm nylon membrane and washed. Dendritic cells were sorted from macrophages by gating on the CD64^−^ population of live GFP^+^ cells followed by bulk RNA sequencing, and analysis with the Tuxedo suite of applications.

### Patient skin and lung explant cultures

For skin explant culture, two adjacent 4 mm punch biopsies were obtained from the right arm of patient SSc6. They were immediately placed in skin media of DMEM containing 4.5 g/L D-glucose, L-glutamine, 0.1% FBS, 100 U/ml penicillin-streptomycin and 2.5 mg/L amphotericin B. Excess subcutaneous fat was carefully removed with iris scissors, then each biopsy specimen was lightly floated epidermis side up exposing it to air in the center of a 12-well tissue culture plate containing 1 ml of skin media alone or with addition of anti-mouse/human Ereg antibody 2.5 μg/ml, then incubated at 37° C in 5% CO_2_ humidified incubator. The media was changed after 2 hours, then on days 2, 5, and 7. Used skin media was stored at −20° C prior to protein and LDH measurements. Pro-COL1A1 and TNC were measured using ELISA kits per manufacturer instructions (Abcam, ab210966 and ab213831). LDH activity was measured with CyQUANT LDH Cytotoxicity Assay Kit per manufacturer instructions (Invitrogen C20300).

For lung explants, human IPF lung (44 year-old hispanic male) was obtained from the National Disease Research Interchange (NDRI), kept cold, and received <24 hours after cross-clamp. A diagnosis of IPF, of 2 years duration, was confirmed by NDRI. While kept cold, tissue was first cut into rough strips, then cut into very thin strips. From there, the tissue was cut into small pieces suitable for culture (roughly 50–100 mg in size). Fragments were cultured in 24 well plates in Dulbecco’s Modified Eagle’s Medium: F-12 Ham’s Nutrient Mixture (DMEM:F-12, Gibco 11039–021) without phenol red, containing L-glutamine, 15 mM HEPES, sodium bicarbonate, penicillin, streptomycin and amphotericin antibiotic-antimycotic solution (Gibco), 50 ug/ml Gentamicin and 1X Insulin-Transferrin-Selenium-Ethanolamine liquid supplement (Sigma). Media changes were performed every 2 days, for a total of 10 days, with replacement of described pharmacologies occurring each media change. After 10 days, culture supernatants were collected, pre-cleared at 4000 X g (4⁰C, 10 mins), transferred to a clean 96 well polypropylene plate and stored at −80⁰C until protein endpoints measured. Measured secreted proteins include: human MCP-1 V-Plex (MesoScale Discovery, K151NND-1), human TIMP-1 (MesoScale Discovery, K151JFC-1) and human pro-collagen 1α1 DuoSet Elisa (R&D systems, DY6220–05). All proteins were assayed and analyzed in accordance with their respective assay product datasheets. After 10 days, lung fragments were harvested and dissociated in 350 μl of RLT buffer containing β-mercaptoethanol using a TissueLyser II (Qiagen) and mRNA isolated with the RNeasy Fibrous mini kit (QiaGen, 74704). Secreted protein results represent 8 individual fragments and gene expression represents 4 individual fragments per treatment group.

### Hydroxyproline Analysis

After euthanasia, the shaved skin or lung was stored at −70° C prior to processing. From the skin, a 2 mm punch biopsy (Accu-Punch) was obtained from the affected area for hydroxyproline quantitation using a Hydroxyproline Assay Kit (Sigma MAK008). Briefly, the 2 mm skin specimen or 3 right lung lobes were boiled in 100 μl or 500 μl, respectively, of 37% hydrochloric acid for 3 hours at 120° C. The sample was then centrifuged for 1 minute at 16,000xg to pellet any remaining hair and debris. From the supernatant, 3 μl was transferred to a fresh microcentrifuge tube and allowed to air dry with the top open at 60° C for approximately 25 minutes. The dried pellet was suspended in 100 μl Chloramine T/Oxidation Buffer mixture for 5–10 minutes, followed by addition of 100 μl diluted 4-(Dimethylamino)benzaldehyde and incubated for 90 minutes at 60° C. The 550 nm absorbance was measured with either BioRad iMark or BioTek Synergy HTX microplate reader.

### Skin Collagenase Assay

Skin collagenase activity was assessed using a fluorometric assay kit per manufacturer’s instructions (Abcam ab234624). Briefly, after euthanasia of experimental mice, a 5 mm punch biopsy of affected skin was obtained. The skin was homogenized in 100 μl cell lysis buffer using a glass Dounce homogenizer and debris pelleted at 16,000 × g at 4° C for 10 minutes. Protein concentration of the supernatant was measured using DC protein assay (Bio-Rad). For the collagenase assay, 25 μl of skin lysate supernatant was mixed with 25 μl collagenase assay buffer in a 96-well glass bottom plate (Cellvis P96–1.5P). Each sample was then mixed with 50 μl collagenase substrate mix and immediately loaded into BioTek Synergy HTX microplate reader to measure fluorescence at Ex/Em 490/520 every minute for one hour. Collagenase activity was calculated from the change in relative fluorescent units per minute from the skin lysate compared to a FITC standard curve and normalized to the skin lysate protein concentration.

### Cell Lines

THP-1 monocytes and human foreskin fibroblasts were purchased from ATCC (TIB-202 and SCRC-1041). CD14^+^ monocytes and CD1c^+^ dendritic cell precursors were isolated from freshly obtained peripheral blood from healthy volunteers using the Human CD14 Positive Selection Kit II from STEMCELL Technologies and CD1c^+^ Human Dendritic Cell Isolation Kit from Miltenyl Biotec per manufacturers’ protocols. Human BMDC were generated by incubating bone marrow from MISTRG6 humanized mice (88, 89) with human GM-CSF (R&D Biosciences 215-GM) 100 ng/ml for 7 days.

### Monocyte and dendritic cell gene expression

Monocytes and dendritic cells were incubated in Gibco Roswell Park Memorial Institute (RPMI) 1640 Medium containing 10% fetal bovine serum (FBS) at 37° C in 5% CO_2_ humidified incubator. They were incubated with cytokines for four hours unless otherwise indicated prior to RNA isolation and cDNA synthesis using the following concentrations: IFNa2 1000 U/mL (Biolegend 592704), TGF-β1 0.64 ng/ml (R&D Biosciences 7754-BH), endothelin-1 100 ng/ml (Abcam ab158332), IL-4 25 ng/ml (R&D Biosciences 6507-IL), and IL-6 100 ng/ml (R&D Biosciences 206-IL). For EREG protein quantification, supernatant from THP-1 monocytes (500,000 per well) incubated with IFNa2 for 4 hours was measured by ELISA (Abcam ab277077). To test the effects of NOTCH ligands, monocytes were incubated in media alone or IFNa2 1000 U/mL for 6 hours at 37° C. During the last 45 minutes, recombinant NOTCH ligands DLL4 (R&D Biosciences 1506-D4) and NOV (R&D Biosciences 1640-NV) each 10 μg/mL were adhered to the bottom of 48 well plates at 37° C for 45 minutes as described (90). The monocytes were pelleted and suspended in fresh media, then transferred to the wells containing NOTCH ligands or media alone.

### Fibroblast gene expression

Human foreskin fibroblasts (HFF) were seeded in Dulbecco’s Modified Eagle Medium (DMEM) containing 1% FBS overnight at 37° C in 5% CO_2_ humidified incubator. The following day, the media was removed and fresh media was added. To test the effects of recombinant human EREG (R&D Biosciences 1195-EP) on NOTCH signaling, 1 μg/ml was added to confluent HFF in media supplemented with ascorbic acid 50 μg/mL overnight prior to RNA extraction and cDNA synthesis. For EGFR ligand expression, sub-confluent HFF were incubated for 48 hours in media alone. For EGFR inhibition, sub-confluent HFF were incubated in media alone or with anti-human EREG neutralizing antibody (R&D Biosciences AF1195) 5 μg/mL for 24 hours prior to RNA isolation and qPCR analysis.

### RNA preparation and Quantitative PCR (qPCR)

RNA was extracted from tissue and cells using RNeasy Mini Plus Kit (Qiagen). Skin and lung tissue were disrupted and homogenized with Qiagen TissueRuptor II in RLT Plus buffer containing β-mercaptoethanol 1:100 dilution and Reagent DX (Qiagen) 1:200 dilution until no remaining intact tissue was visible, approximately 30–60 seconds. cDNAs were developed using Maxima H Minus Reverse Transcriptase 10 U/μl supplemented with 0.5 mM dNTPs and 25 ng/μl Oligo d(T)_20_. Primers were purchased from Sigma-Aldrich and sequences are listed in Tables S3 and S4. Relative quantification of gene expression using SYBR Green was measured with CFX384 or CFX96 Touch Real-Time PCR Detection System (Bio-Rad). Gene expression was normalized to the housekeeping gene UBC (91) or PPIA (lung explants) and relative expression was calculated using the 2^−ΔΔCt^ method (92).

### Statistical analysis

Differential gene expression analysis of scRNA-Seq data was performed using Cell Ranger and Loupe Cell Browser software (10x Genomics), which uses a variant of the negative binomial exact test from sSeq and the asymptotic beta test in edgeR depending on sample size (93, 94). Statistical analysis and graphs of other data were generated using GraphPad Prism v9. Pairwise comparisons were analyzed using two-tailed Student t test and multiple comparison with one-way analysis of variance (ANOVA). For all graphs and heatmaps, *P<0.05, **P<0.01, ***P<0.001, ****P<0.0001.

## Supplementary Material

data file S2

data file S3

main supplementary materials

reproducibility checklist

data file S1

## Data Availability

The scRNA-seq data generated in this study have been deposited in the Gene Expression Omnibus under accession number GSE214088. All other data needed to support the conclusions of the paper are present in the paper or the Supplementary Materials.
