# Supplementary material for: Epiregulin is a dendritic cell-derived EGFR ligand that maintains skin and lung fibrosis
Source: Sci Immunol. Author manuscript; Available in PMC 2023 Jan 14. (PMC9840167; doi:10.1126/sciimmunol.abq6691)
Supplement: main supplementary materials [file NIHMS1861570-supplement-main_supplementary_materials.docx]

**
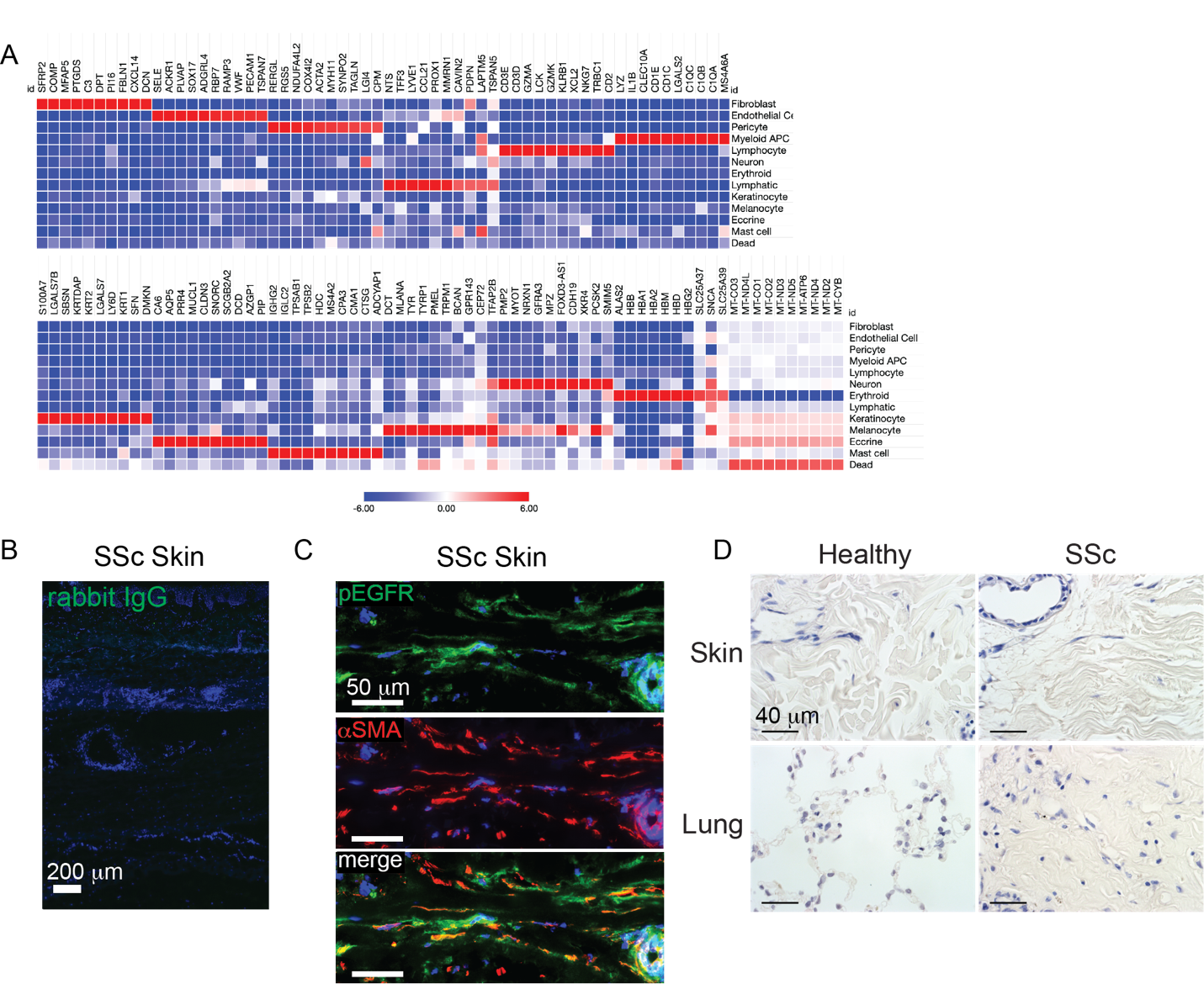
**

**Supplementary Fig. 1**. **Cell cluster signature genes and immunostains of SSc skin**. (A) Major cell clusters and signature genes. Heatmap of log_2_(fold change) of gene expression of top 10 upregulated genes per cluster in aggregated scRNA-Seq data of healthy controls and SSc samples. (B) SSc skin stained with rabbit IgG isotype control antibody. (C) SSc skin stained for pEGFR Tyr-1068 and α-SMA. (D) Healthy and SSc skin and lung stained with rabbit IgG isotype control antibody. Images are 4x (B) and 40x (C, D) magnification.


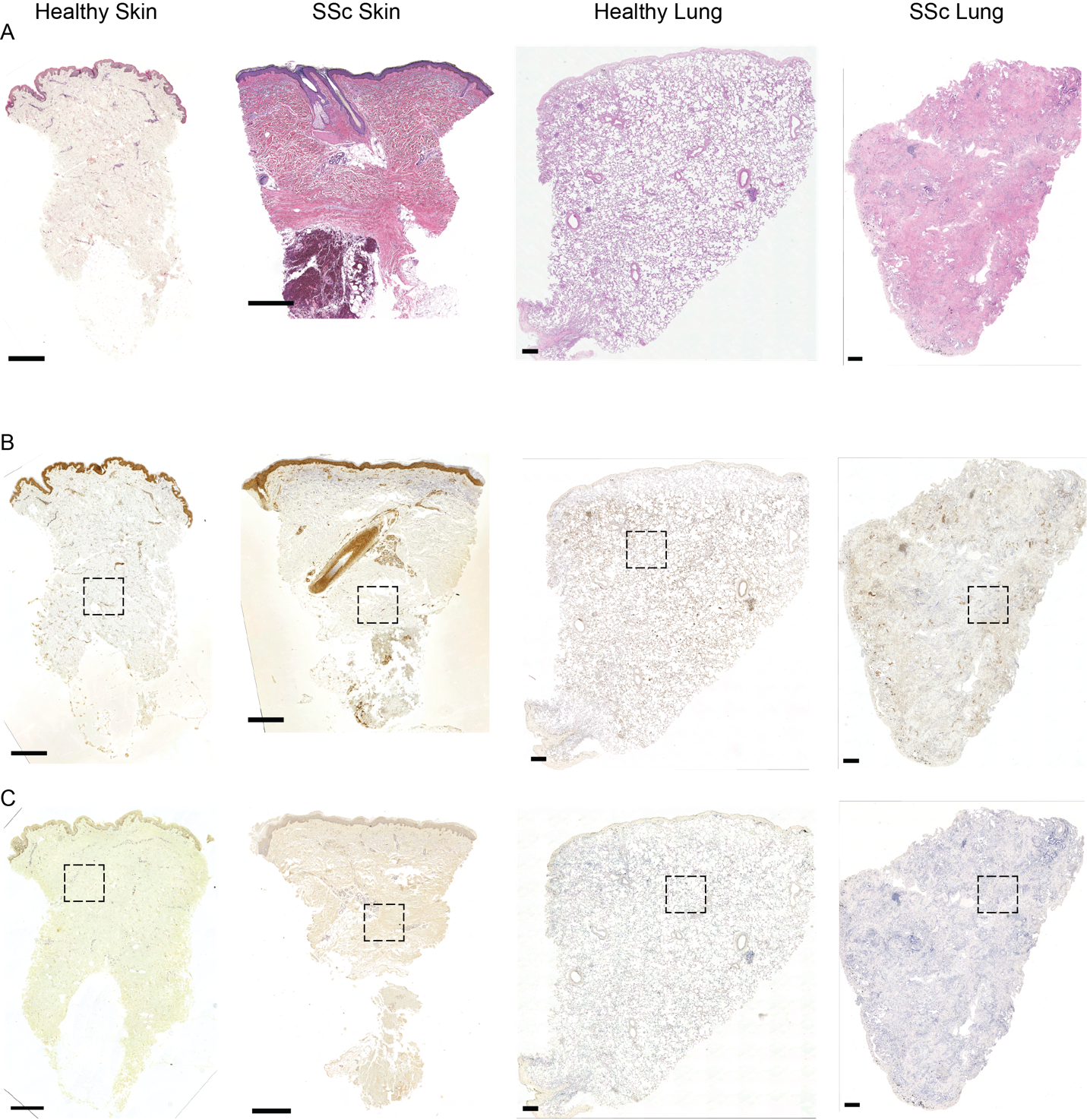


**Supplementary Fig. 2. Healthy and SSc skin and lung low power images.** Healthy and SSc skin and lung were stained for histology by hematoxylin and eosin (A), pEGFR Tyr-1068 (B), and EREG (C). Dashed boxes label the location of the high-power images of the skin shown in Fig. 1 and 2. Slides were imaged with a Keyence BZ-X800 microscope at 10x magnification and stitched together using their software. Scale bars are 500 microns.


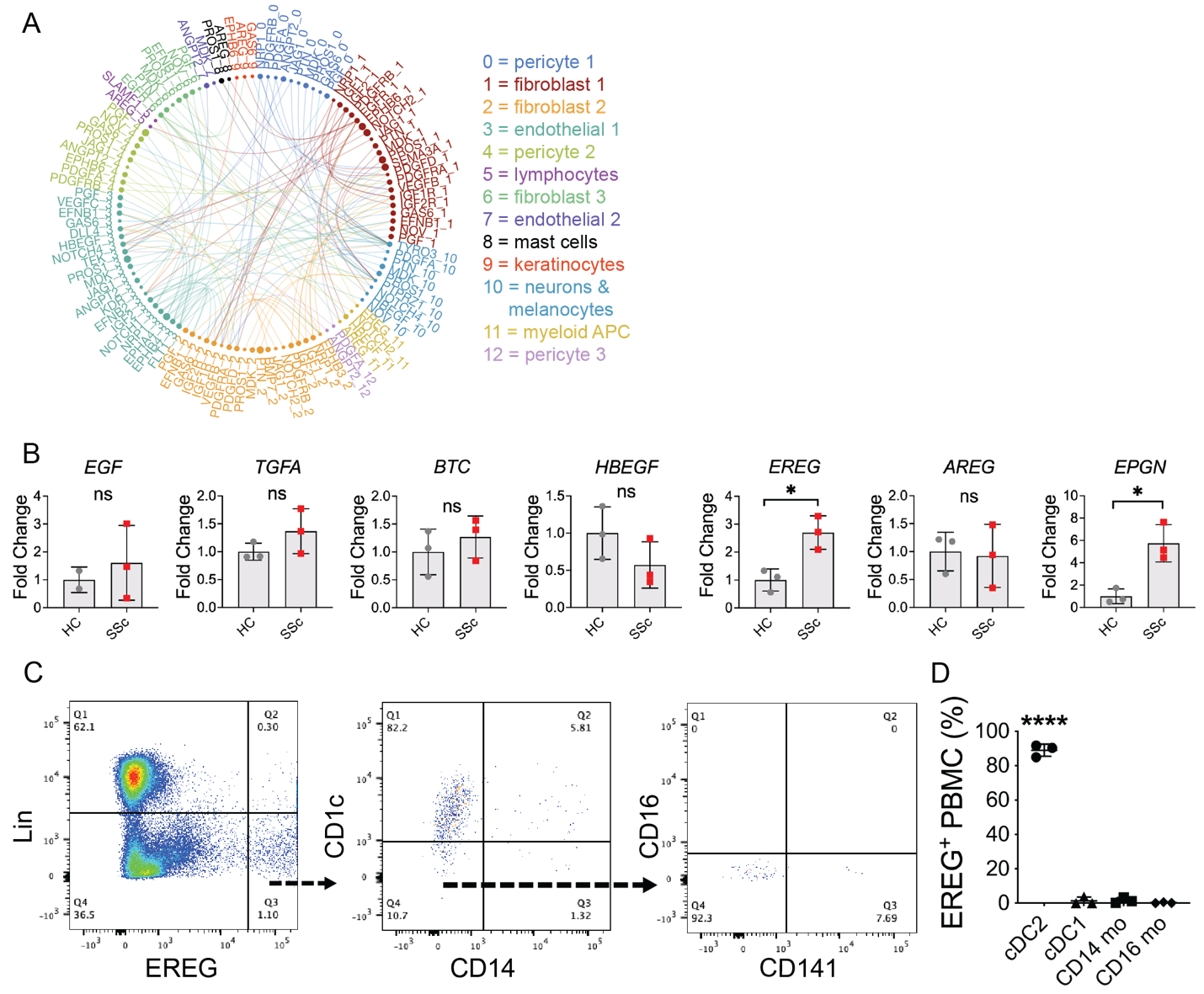


**Supplementary Fig. 3. EREG^+^ dendritic cell marker expression and immunophenotyping.** (A) Diagram of enriched receptor-ligand growth factor pairs in SSc skin. (B) Relative expression of EGFR ligands from whole tissue of healthy and SSc frozen skin (n=3 per group). (C) PBMC isolated from healthy volunteers were stained for EREG along with adaptive and myeloid cell markers and analyzed by FACS. Gating shows live CD45^+^ cells, followed by EREG^+^ lin^-^ (CD3/CD20/CD66b/NKp46) cells to exclude T cells, B cells, granulocytes, and NK cells. Data is representative from 2 healthy volunteers. (D) Quantification of dendritic cell subsets among EREG^+^ cells with cDC2 including all CD1c^+^ cells and cDC1 by CD141^+^ staining. Data are means ± SD (ns, not significant, *P < 0.05, ****P<0.0001) analyzed with unpaired two-tailed Student t test (B) and one-way ANOVA with Tukey multiple-comparisons test (D).

**
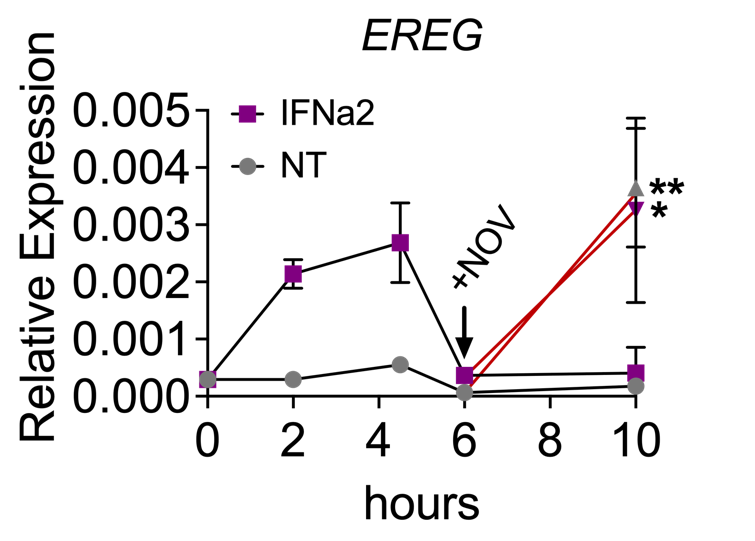
**

**Supplementary Fig. 4. NOTCH ligand NOV induces *EREG* expression in BMDC.** *EREG* relative expression by BMDC treated with IFNα2 prior to exposure to NOTCH ligand NOV (n=3-4 per time point in each group).


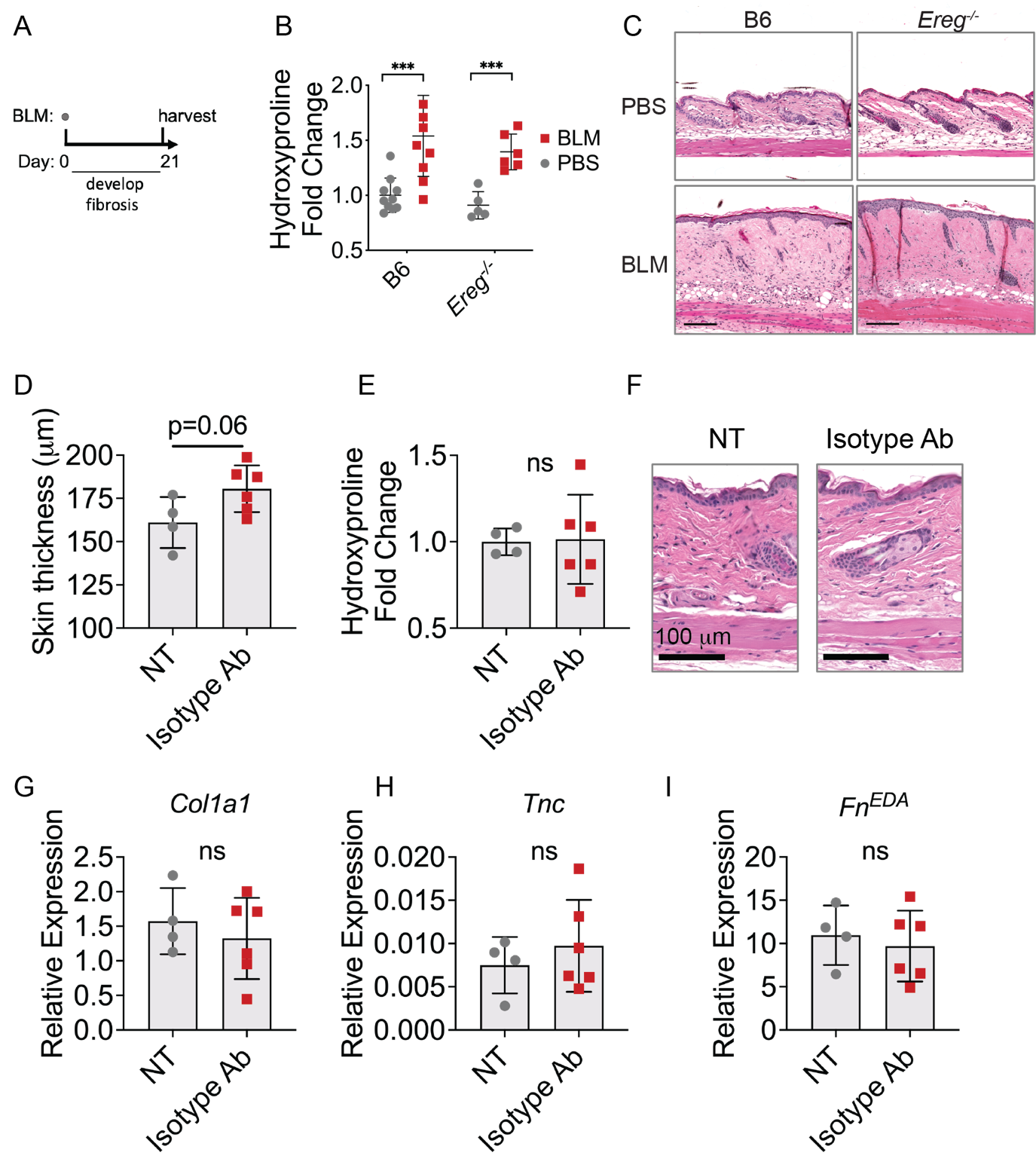


**Supplementary Fig. 5. *Ereg*^-/-^ mice develop skin fibrosis similar to wild type.** Diagramed in (A), cohorts of B6 and *Ereg*^-/-^ mice were injected with bleomycin subcutaneously and 21 days later analyzed for hydroxyproline (B) and histology (C). (D-I) B6 mice were injected with bleomycin subcutaneously and treated with isotype antibody corresponding to Ereg Ab used in Figure 5 for two weeks and analyzed for skin thickness (D), hydroxyproline (E), histology (F), and ECM gene expression (G-I).

**Supplementary Table 1.** Select clinical characteristics of the patients in this study.

| **Sample** | **Age (years)** | **Sex (M/F)** | **Ethnicity** | **Biopsy Site** | **Duration (year)** | **Past Medical History** | **Other organ involvement** | **Autoantibodies** | **Immunomodulating Medications** |
| --- | --- | --- | --- | --- | --- | --- | --- | --- | --- |
| SSc1 | 60 | M | W | Right dorsal hand | 0.33 |  | renal | RNA Pol III | none |
| SSc2 | 61 | M | W | Thigh | 22 |  | none | negative serologies | ECP |
| SSc3 | 39 | M | W | Left forearm | 4 |  | interstitial lung disease esophageal dysmotility | ANA 1:2560 | Bosentan ECP |
| SSc4 | 57 | F | W | Forearm | 2 | inflammatory bowel disease | inflammatory arthritis | ANA 1:320 RNA Pol III CCP | MMF phototherapy prednisone HCQ |
| SSc5 | 47 | F | W | Chest | 10 |  | interstitial lung disease pulmonary arterial hypertension | ANA 1:320 SSA RNP | MMF HCQ sildenafil |
| SSc6 | 54 | F | W | Right forearm | 0.75 |  | interstitial lung disease  GERD | ANA 1:320  RNA Pol III | MMF |
| SSc7 | 71 | F | W | Right  forearm | 4 | atopic dermatitis | interstitial lung disease  GERD | ANA 1:640  Centromere | dupilumab |
| Healthy1 | 24 | F | W |  |  | none |  |  |  |
| Healthy2 | 35 | M | W |  |  | dermographism |  |  |  |
| Healthy3 | 54 | M | W |  |  | none |  |  |  |
| Healthy4 | 52 | M | W |  |  | BCC |  |  |  |
| Healthy5 | 38 | M | W |  |  | none |  |  |  |
| Healthy6 | 24 | F | W |  |  | none |  |  |  |
| Abbreviations: W, white; M, male; F, female; GERD, gastroesophageal reflux disease; ECP, extracorporeal photopheresis; MMF, Mycophenolate mofetil; HCQ, hydroxychloroquine; BCC, basal cell carcinoma | | | | | | | | | |

**Supplementary Table 2. List of antibodies.**

| **Antibody** | **Company** | **Catalogue Number** | **Clone** | **Dilution or Concentration** |
| --- | --- | --- | --- | --- |
| Rabbit anti-human/mouse EGFR (phospho Y1068) | Abcam | ab40815 | EP774Y | human 1/400, mouse 1/800 |
| Rabbit IgG isotype control | Abcam | ab172730 | EPR25A | 1/400 |
| Anti-human epiregulin antibody | R&D Biosciences | AF1195 |  | 5 mg/mL |
| Rat anti-human procollagen type 1 | Abcam | ab64409 |  | 1/100 |
| Rat anti-mouse/human epiregulin antibody | R&D Biosciences | MAB1068 | 189611 | 10 mg/kg per mouse |
| Mouse IgG2a isotype control | Bio X Cell | BE0085 |  | 10 mg/kg per mouse |
| Anti-mouse IFNAR-1 antibody | Bio X Cell | BE0241 | MAR1-5A3 | 1.67 mg per mouse |
| Anti-human CD146 | Biolegend | 342011 | SHM-57 | 1/100 |
| Anti-human CD26 | Biolegend | 302718 | BA5b | 1/100 |
| Rabbit anti-human alpha smooth muscle actin | Abcam | ab202296 |  | 1/100 |
| Rabbit anti-human FCN1 | Abcam | ab223712 |  | 1/40 |
| Rabbit anti-human/mouse Fcn1 | Invitrogen | ABS 036-05-02 |  | 1/40 |

**Supplementary Table 3. List of primers used for quantitative PCR of human genes.**

| **Gene** | **Forward Primer Sequence** | **Reverse Primer Sequence** |
| --- | --- | --- |
| UBC | CGTCACTTGACAATGCAG | TGTTTTCCAGCAAAGATCAG |
| COL1A1 | GCTATGATGAGAAATCAACCG | TCATCTCCATTCTTTCCAGG |
| FN1^EDA^ (*55*) | TAAAGGACTGGCATTCACTGA | GTGCAAGGCAACCACACTGAC |
| TNC | GTGGGATCCTCTAGACATTG | GTGATCTCTCCCTCATCTTC |
| EGF | CAGTTGATCCAGTAGAAAGG | TGTACTGAATCCAAAACAGC |
| TGFA | AGAAACAGTGGTCTGAAGAG | ATTACAGGCCAAGTAGGAAG |
| BTC | CAGAAGTCCTGAAACTAATGG | CAATGTAGCCTTCATCACAG |
| HBEGF | GCTTATATACCTATGACCACAC | GTACCTAAACATGAGAAGCC |
| EREG | GTTCAGACAGAAGACAATCC | GACTCATGTCCACCAGATAG |
| AREG | AAAGAAAGAAAAAGGGAGGC | CATTTGCATGTTACTGCTTC |
| EPGN | GCAAGCTGACAACATAGAAG | GCAGTAACTGTTATGATCTTCC |
| DLL4 | GTTACACAGTGAAAAGCCAG | CTCTCCTCTGATATCAAACAC |
| NOV | TAGAAGTCTCTGACTCAAGTG | CCTTTCTTATCTGTTGGCTG |
| JAG1 | ACTACTACTATGGCTTTGGC | ATAGCTCTGTTACATTCGGG |
| NOTCH1 | AAGATATGCAGAACAACAGG | TCCATATGATCCGTGATGTC |
| NOTCH2 | GATGAATGATGGTACTACACC | CAGATTTTCCATGGTCATCC |
| NOTCH3 | CTACAATGGTGATAACTGTGAG | CAGTCATCCTCATTAATCTCG |
| NOTCH4 | GTCCCCCAGGTTTCATAG | ACTCATCCACATCTTCGG |
| HES1 | GCCTATTATGGAGAAAAGACG | CTATCTTTCTTCAGAGCATCC |
| HES4 | AAAACCCTCATCCTGGAC | GTGTCTCACGGTCATCTC |

**Supplementary Table 4. List of primers used for quantitative PCR of mouse genes.**

| **Ubc** | **GAGACGATGCAGATCTTTG** | **ATGTTGTAGTCTGACAGGG** |
| --- | --- | --- |
| Col1a1 (*93*) | GCTCCTCTTAGGGGCCACT | CCACGTCTCACCATTGGGG |
| Fn1^EDA^ (*55*) | AGATGCAGCAGATCCGCAT | GTTCTTGCCCATCAGCACC |
| Tnc | CAAAGAGACCTTCATCACAG | GGCTCTTTGGAACATCTATC |
| Egf | GTACTCTTGGGTGTGAAAAC | CAAGTTCGTGACATTGTTTC |
| Tgfa | CTCTGAGACAGTGGTCTG | GTCCCAAGATGCCTTTTC |
| Btc | AGACCAATATTGCTTAACGG | AACAAGCAACACACAGTTAC |
| Hbegf | GAGCTATAGGAACCTTCAGAG | ACTGGAAATGAATGAAGACG |
| Ereg | AATTCTGCAGATGTGAAGTG | TATTCTTTGCTCAAGGGTTG |
| Areg | GACATGCAATTGTCATCAAG | GACAAAGATAGTGACAGCTAC |
| Epgn | ATAGAAGAACCTGTAGCTCTG | CCGTATAACCAGTAAAGCATC |
